# Cardiac phase modulates behavior and response related lateralization in visual spatial conflicts during change detection

**DOI:** 10.1162/IMAG.a.1150

**Published:** 2026-02-27

**Authors:** Leon von Haugwitz, Edmund Wascher, Mauro F. Larra

**Affiliations:** Department of Ergonomics, IfADo – Leibniz Research Centre for Working Environment and Human Factors, Dortmund, Germany

**Keywords:** visual spatial attention, event related lateralization, heart-brain, cardioafferent, conflict task, stimulus-response-paradigm

## Abstract

The brain is in continuous bidirectional exchange with the heart, receiving and sending signals that not only sustain physiological regulation but also shape perceptual and higher-order cognitive processes. While attentional selection has long been conceptualized as a mechanism for guiding behavior through external sensory input, it remains unclear how afferent signals originating from the heart are integrated into this process. To investigate how such cardioafferent signals interact with attentional selection, we employed a change detection task synchronized to the cardiac cycle which induced perceptual conflicts by pairing task-relevant luminance changes with more salient orientation changes in the opposing hemifield. Seventy-six participants were pseudorandomized into two groups exposing them to either repeated cold pressor tests (CPT, N = 51) or a warm water control condition (N = 25) to probe cardiovascular drivers of cardiac cycle effects. Cardiac phase systematically modulated change detection: systolic onsets increased errors in spatial-conflict changes, biasing responses toward a salient distractor, and increased misses for isolated luminance changes. EEG lateralization in a central–posterior cluster implicated altered premotor response encoding rather than early sensory gating (change positivity, N1pc, N2pc) as the locus of these effects. Although exposure to the CPT elevated blood pressure and heart rate, we found no robust group-level modulation of the cardiac-phase effect; instead, individual differences in heart rate responses to the CPT as well as an interaction of systolic blood pressure and heart rate variability predicted the magnitude of spatial-conflict phase effects in the CPT group. Together, these results demonstrate that phasic bodily signals can bias visuomotor selection during perceptual conflicts and change detection.

## 1 Introduction

Attention shapes our subjective experience by selectively enhancing relevant information while suppressing distractions, guiding perception and action in a world of constant sensory input (Beck & Kastner, 2009; Carrasco, 2011; Chica et al., 2013; Corbetta & Shulman, 2002; Desimone & Duncan, 1995). Such top-down control is mediated through fronto-parietal networks that bias neural activity in early sensory areas thereby enhancing attended and suppressing distracting stimuli (Beck & Kastner, 2009; Chelazzi et al., 1993, 2001; Connor et al., 2004; Fries et al., 2001; Luck et al., 1997; Motter, 1993; Reynolds & Desimone, 2003; Reynolds et al., 1999). However, stimuli may also capture attention in a bottom-up fashion and ultimately attentional selection arises from the interplay of top-down and bottom-up factors such as stimulus salience (e.g., brightness, motion, novelty, affective quality). Beyond external stimuli, our brain constantly receives signals from peripheral organs. It is now well established that these internal bodily signals interact with the processing of external stimuli (Al et al., 2020; Azevedo et al., 2017; Critchley & Harrison, 2013; Engelen et al., 2023; Kluger et al., 2023), raising the question how this might affect attentional selection and corresponding behavioral responses.

One of the most prominent sources of these bodily signals is the heart: as the first organ to develop, it initiates regular contractions weeks before neural control emerges subsequently sending afferent input to the brain while adapting its rhythm to environmental demands throughout our whole life (Filosa & Sawamiphak, 2023; Valenza et al., 2025). These afferents primarily arise from baroreceptors, stretch-sensitive mechanoreceptors essential for blood pressure and heart rate regulation via the baroreflex (Mancia & Grassi, 1995; Stauss, 2002). Located in the aortic arch and carotid arteries, baroreceptors fire in response to both tonic and phasic blood pressure changes, peaking during systole when arterial walls are stretched and reaching a minimum during diastole (Rau & Elbert, 2001). Baroreceptor signals ascend via the glossopharyngeal and vagus nerves to the nucleus of the solitary tract. From there, they converge with other visceral inputs before projecting to cortical regions such as the insula and anterior cingulate (Critchley & Harrison, 2013). At the same time, heartbeats also cause palpable somatosensory sensations that may further contribute to interactions with external signal processing (Macefield, 2003; Sophie et al., 2021). Moreover, the brain may have developed endogenous attentional mechanisms resulting from the predictability of heartbeat-related signals throughout life. It has been proposed that these could act akin to oscillatory attentional filters which may also exert a phase-dependent effect on perception (Al et al., 2020, 2021; Barrett & Simmons, 2015; Saltafossi et al., 2023; Seth et al., 2011). Together, these pathways provide a functional basis that allows for widespread effects potentially influencing attentional selection via both, bottom-up and top-down mechanisms.

Indeed, it has been shown that detection and awareness of basic visual stimuli is inhibited when stimuli are presented concurrent with heartbeats, during ventricular systole, that is, at times of increased cardioafferent activity (Edwards et al., 2007; Leupin & Britz, 2024; Ren et al., 2022a, 2024; Salomon et al., 2016, 2018; Walker & Sandman, 1982). These effects have mostly been attributed to well-known inhibitory effects of baroreceptors (Dworkin et al., 1994; Lacey & Lacey, 1978). More recent account propose that they are driven by modulations of perceptual gain due to the co-activation of interoceptive and multimodal integration areas when visual and bodily signals are processed simultaneously (Leupin & Britz, 2024; Skora et al., 2022). Interestingly, salient affective stimuli (Azevedo et al., 2018; Critchley & Garfinkel, 2015; Garfinkel et al., 2014; Park et al., 2013; Yang et al., 2023) form an exception to such perceptual inhibition and in more complex visual scenes selection efficiency was found to be improved at systole (Marshall et al., 2024; Pramme et al., 2014, 2016). This suggests beneficial effects of inhibition in suppressing distractors and prioritizing task-relevant input. On the other hand, cardiac activity may also exert a more direct effect on behavioral outcomes. Response inhibition (Leganes-Fonteneau et al., 2025; Makowski, Sperduti, et al., 2019; Rae et al., 2018; Ren et al., 2022b; Yang et al., 2023), initiation of voluntary actions (Kunzendorf et al., 2019; Mussini et al., 2024; Palser et al., 2021), as well as motor imagery (Lai et al., 2024) and excitability (Al et al., 2023; Paci et al., 2024) have all been shown to vary across the cardiac cycle, whereas in some contexts stimuli presented during systole triggered automatic reactions, an effect dependent on sensorimotor compatibility (Larra et al., 2020; von Haugwitz et al., 2024). Thus, it remains unclear how perceptual inhibition across the cardiac cycle modulates biased competition and if those effects interact with top-down attentional selection, particularly when distractors compete with task-relevant targets.

To investigate this, we employed EEG recordings during a change detection task (Wascher & Beste, 2010) that manipulated target salience and introduced spatial conflicts between task-relevant and more salient but task-irrelevant changes. Specifically, task-relevant unilateral luminance changes (LUM) were paired either with a more salient orientation change at the same location (LOU) or in the opposite hemifield (LOB), while isolated orientation changes served as catch trials (ORI). Change onsets were synchronized to the ECG to occur during ventricular systole or diastole. The symmetrical lateral stimulus arrangement enabled analysis of event-related lateralization (ERL) at posterior sites, utilizing the contralateral mapping of visual pathways to isolate lateralized processing from early to late stages. Early ERLs in the P1 range, termed change positivity (P1pc), index change detection (Busch et al., 2010; Verleger et al., 2012). The subsequent N1pc reflects the initial allocation of attention toward the most salient stimulus (Schneider & Wascher, 2013; Schneider et al., 2012a, 2012b; Wascher & Beste, 2010). When this allocation is misdirected by a salient distractor, the N1pc is typically followed by an N2pc toward the task-relevant target, reflecting top–down guided selection and distractor suppression (Eimer, 1996; Hickey et al., 2009; Luck & Hillyard, 1994; Woodman & Luck, 1999, 2003). To disentangle these stimulus- from response-related effects mentioned above, we further applied temporal EEG decomposition of ERLs using residue iteration decomposition (RIDE; Ouyang et al., 2015a, 2015b). Finally, we analyzed change evoked midfrontal theta oscillations as a correlate of conflict processing and cognitive control (Asanowicz et al., 2021; Cohen, 2014; Muralidharan et al., 2023; Tollner et al., 2017), as well as lateralized power spectra in the alpha frequency range at posterior sites (LPS) as an additional index of attentional target processing dissociated from the N2pc (Asanowicz et al., 2021; Bacigalupo & Luck, 2019; Thut et al., 2006; Van der Lubbe & Utzerath, 2013).

A second aim of this study was to shed light on the pathways and cardiovascular drivers involved in the observed cardiac cycle effects. To this end, we repeatedly employed the Cold Pressor Test (CPT), a well-established protocol to non-invasively increase blood pressure via peripheral vasoconstriction. This leads to an increase in baroreceptor loading, consequently altering heartbeat rhythms and sympathovagal control by activation of the baroreflex (Hines & Brown, 1936; Houben et al., 1982; Lovallo, 1975; Victor et al., 1987). As mentioned above, inhibited perceptual performance during systole has been associated with variation in baroreceptor firing affecting cortical excitability (Dworkin et al., 1994; Lacey & Lacey, 1978; Leupin & Britz, 2024). Cardiac cycle effects have also been linked to heart rate variability (HRV). Al et al. (2020) found that individuals with lower HRV (RMSSD) were more likely to miss near-threshold tactile stimuli during systole. Within the interoceptive predictive coding framework, this has been interpreted as indication that lower variance in interoceptive signals enhances the precision of sensory attenuation to shield exteroceptive processing from internal noise. However, other studies did not find a clear association between HRV and cardiac cycle effects (e.g., Al et al., 2021; Azevedo et al., 2018; Kunzendorf et al., 2019; Rae et al., 2018). Moreover, interindividual differences in sensitivity to baroafferent signals are an important factor to consider and may be revealed by cardiovascular regulation patterns during the CPT (Hines & Brown, 1936; Lovallo, 1975): Exposure typically elicits an initial rise in blood pressure and heart rate (HR) whereby at prolonged exposure the baroreflex reduces HR in response to the increase in blood pressure (Cui et al., 2002; Frank & Raja, 1994; Houben et al., 1982; Mancia & Grassi, 1995; Stauss, 2002; Victor et al., 1987).

We conducted a between-subjects study with pseudorandomized group allocation, oversampling the CPT group to increase statistical power. As prior work on cardiovascular manipulations has not yielded group-level effects (c.f. Mussini et al., 2024; von Haugwitz et al., 2024), we additionally performed regression analyses within the CPT group to account for the interindividual variability in cardiovascular responses to the CPT. To probe the previously suggested cardiovascular drivers, we used systolic blood pressure (SBP) during experimental blocks as a proxy for baroreceptor signal strength during systole (c.f. Dworkin et al., 1994; Lacey & Lacey, 1978; Leupin & Britz, 2024), RMSSD during experimental blocks as a measure of HRV (c.f. Al et al., 2020), and SBP and HR responses to the CPT as indicators of homeostatic regulatory function (c.f. Hines & Brown, 1936; Lovallo, 1975).

Our central hypothesis is that effects of cardioafferent traffic are not uniformly inhibitory but will instead affect performance depending on the type of visual change. Specifically, we expect that early sensory attenuation during systole will impair detection of luminance changes, in particular when presented in isolation (LUM). By contrast, such effects should be reverted if paired with ipsilateral orientation changes (LOU) and for unilateral orientation changes (ORI), as highly salient stimuli are often less susceptible to systolic inhibition. When luminance and orientation changes occur simultaneously in opposite hemifields (LOB), improved performance during systole would indicate enhanced distractor suppression, whereas reduced performance would suggest stronger salience-driven processing. Finally, we expect that blood pressure increases due to CPT exposure will modulate cardiac phase dependent effects. In particular, enhanced phase-dependent effects with responses characterized by rising SBP coupled with increased vagal control of heart rate (HR) and heart rate variability (HRV) would be indicative of an association with baroreflex activation and thus be supportive of a baroreceptor driven mechanism.

## 2 Methods

### 2.1 Sample

A total of 81 healthy individuals initially participated in the experiment. Five participants were excluded from further analysis: three due to performance below chance level and two due to excessive artifacts in their EEG/ECG recordings. The final sample comprised 76 participants (female = 36, male = 40, age = 24.55 ± 4.19 years), all of whom were right-handed (laterality quotient (LQ): *M* = 94.13 ± 10.81) according to the Edinburgh Handedness Inventory (Oldfield, 1971). Participants were pseudorandomized into either the CPT (N = 51, female = 26, male = 25, age = 23.98 ± 3.72 years, LQ = 93.54 ± 11.19) or control group (N = 25, female = 11, male = 14, age = 25.04 ± 5.03 years, LQ = 93.49 ± 10.06) to achieve double the sample size in the experimental group. Participants in the CPT and control group did not significantly differ in age (*t*(37.3) = -0.94, *p* = .356, *d* = -0.15, BF_10_ = 0.4) or sex (χ^2^(1) = .03, *p* = .867, *w* = 0.02, BF_10_ = 0.29) or LQ (*t*(52.3) = 0.1, *p* = .918, *d* = 0.01, BF_10_ = 0.25). An a priori power analysis was performed in G*Power 3.1 (Faul et al., 2007) for a repeated-measures within–between interaction to determine the sample size for the planned design which resulted in a sample size of 75 participants. To adjust for increased variance, due to the CPT, we modeled GROUP with three levels (control + two CPT groups). The PHASE × CHANGE cells were approximated as a single within factor with 8 measurements (f = 0.15; α = 0.05; Power = 0.95; Groups = 3; Measurements = 8; r = 0.60; ε = 0.80, actual computed power = 0.96).

The sample consisted mostly of university students, recruited via social media platforms and a university participant pool. Compensation was provided in the form of either course credit or monetary payment (13.50 € per hour). Prior to the experiment, a screening interview was performed to ensure subjects fitted the inclusion and exclusion criteria. Inclusion criteria required participants to be between 18 and 35 years of age, have a body mass index between 19 and 25, and be in good health during the experiment and the preceding 2 weeks. Exclusion criteria were the use of medication or drugs within 2 weeks of the study (with the exception of nonprescription drugs such as aspirin), any acute or chronic medical conditions (in particular any indication of cardiovascular conditions, arrythmias, arterial hypertension, Raynaud’s disease, history of syncope), family history of hypertension, or a history of cerebral or aortic aneurysms or injuries. Individuals with heightened sensitivity to cold or oral health complaints were also excluded. Participants were instructed to abstain from consuming caffeinated or alcoholic beverages, engaging in physical exercise, and eating in the 2 hours prior to their appointment. The study received ethical approval from the IfADo ethics committee and adhered to the Declaration of Helsinki. Informed written consent was obtained from all participants after they were fully briefed about the procedure and their right to withdraw from the experiment at any point.

### 2.2 Procedure

#### 2.2.1 General procedure

Participants were seated in a dimly lit room in a comfortable chair, where EEG and ECG electrodes, and a blood pressure cuff were attached. After setup, participants first completed a training block of the experimental paradigm to familiarize themselves with the task. This was followed by a 10-minute resting phase.

**Fig. 1.**
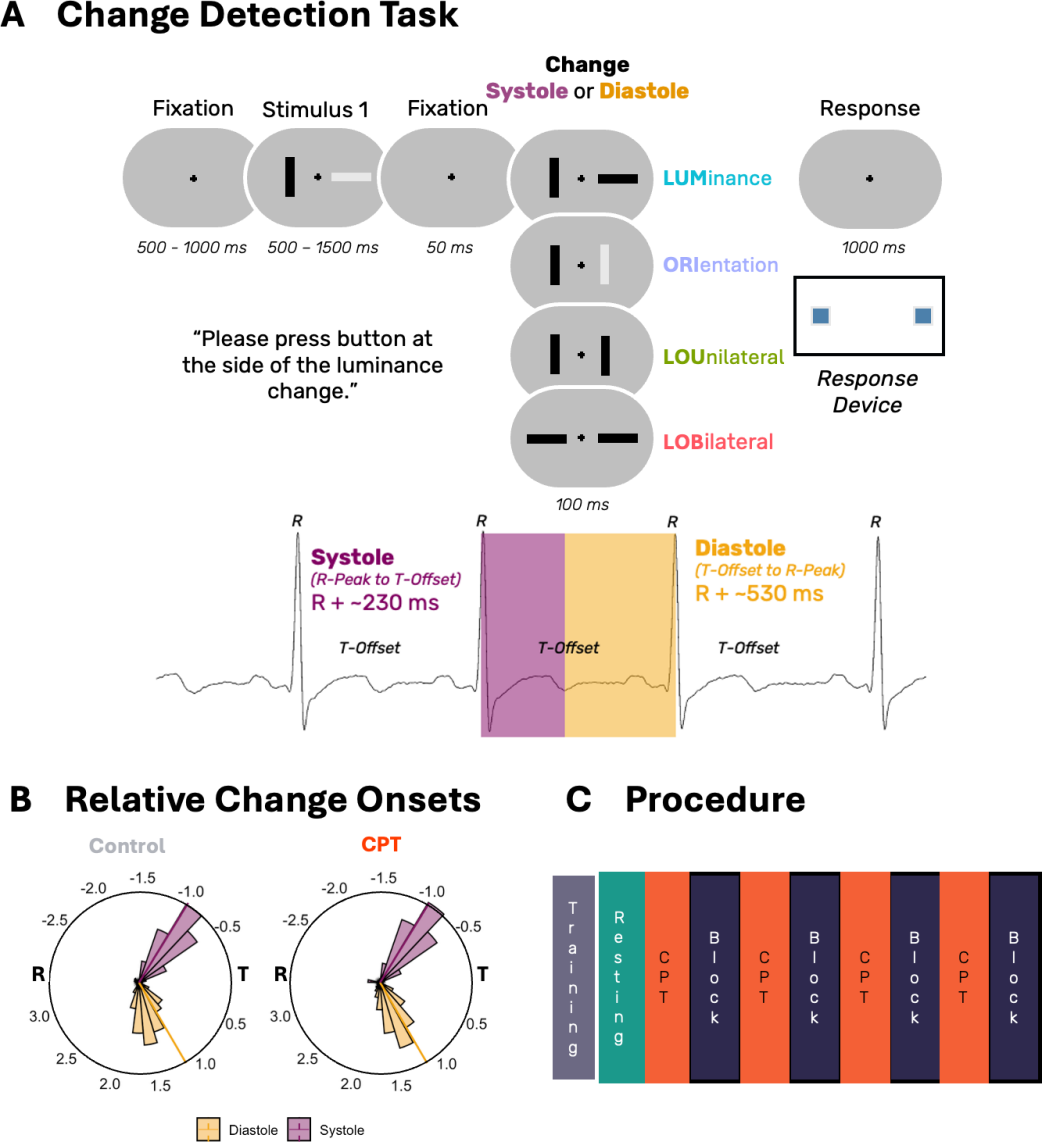
Schematic of change detection paradigm (A) and change onsets expressed as cardiac angle by group, that is, change onsets (expressed in radians, see methods section) relative to R-Peak (R) and offset of the T-Wave (T, B) as well as the experimental procedure (C).

Subsequently, the first cold pressor test (CPT) or warm water control condition was started, immediately followed by the first experimental block. This procedure (CPT-Block) was repeated three additional times (see Fig. 1C).

All experiments were conducted between 1:00 PM and 4:00 PM, participants were not informed in advance about the condition scheduled for their session.

#### 2.2.2 Experimental task and apparatus

Participants performed a change detection paradigm (Wascher & Beste, 2010) where they had to detect the location of a luminance change occurring between two subsequently presented visual frames (see Fig. 1). A fixation cross was presented centrally on screen (viewing distance 80 cm) throughout the experiment . In each trial, the first display comprised two bars (61 px × 25 px, 1.34° × 0.55°, ratio: 1:1.7) presented to the left and right of the fixation cross for a jittered duration (500–1500 ms). The bars varied in luminance and could be either darker (RGB: 21, 21, 21) or brighter (RGB: 104, 104, 104) than the background (RGB: 73, 73, 73). Their orientation was either vertical or horizontal.

Following an interstimulus interval of 50 ms, a second frame with the same layout appeared for 100 ms. Depending on trial condition, four types of feature changes occurred between frames with equal probability: changes in luminance (LUM), changes in orientation (ORI), simultaneous changes in both features at the same location (LOU), or at different locations (LOB). Following this second frame, participants needed to indicate where the change in luminance (if any) occurred.

Stimulus features across both frames were randomly intermixed, ensuring that all combinations of luminance and orientation appeared with equal frequency. This variability ensured that participants could not predict feature changes based on the initial frame, thus requiring active processing to perform the task. Responses were registered via a square response box sized 30 cm × 13 cm equipped with two buttons located on the left and right side. The response box was positioned on a table 30 cm in front of the participant’s midsagittal plane. Participants were instructed to respond as quickly and accurately as possible with the corresponding index finger.

Change onsets were presented time-locked to the cardiac cycle, either during systole (onsets were centered at 230 ms) or diastole (centered at 480 ms) after the R-wave peak. Change onsets were jittered using a Gaussian distribution centered at the target latency (e.g., 230 ms for systole), with a maximum deviation of ±30 ms. Stimulus timing was managed using E-Prime 3.0 (PST Software, Inc.), with synchronization achieved through TTL pulses sent from an AccuSync 72 ECG monitor (AccuSync Medical Research Corporation) via serial interface. Figure 1B displays the actual stimulus-change onsets in radians, representing the change onset in a circularized cardiac cycle. Cardiac angle was computed following the procedure described in by Sherman et al. (2022). Briefly, the calculation uses each change onset’s latency relative to the preceding R-peak, the offset of the T-wave, and the duration of the corresponding interbeat interval (IBI). Onsets occurring before the end of the T-wave, that is, during systole are expressed as a proportion of the R-to-T interval and assigned a negative phase value. Onsets occurring after the T-wave are expressed as a proportion of the remaining T-to-R segment. These normalized proportions are then converted into radians, yielding a continuous circular representation of event timing across the cardiac cycle. We verified the accuracy of R-peak detection by comparing the AccuSync device’s triggers with offline R-peak detection using R-DECO which showed a very high accuracy of the online detection (Percentage trials which had to be recoded due to false cardiac phase onset = 0.03 ± 0.19%). Average delay of the change onset to the previous R-Peak was 223.7 ± 15.32 ms for systole and 523.58 ± 15.3 ms for diastole trials which did not significantly differ between groups for systole (CPT = 223.49 ± 15.89 ms, Control = 224.2 ± 15.32 ms, *t*(70.93) = -0.02, *p* = .816, *d* = -0.03, BF_10_ = 0.26) or diastole onsets (CPT = 523.41 ± 15.86 ms, Control = 523.92 ± 14.12 ms, *t*(70.93) = -0.07, *p* = .947, *d* = -0.007, BF_10_ = 0.25).

Participants completed a training block of 60 trials followed by four experimental blocks of 256 trials each. Within each block, trials were balanced across change condition, frame 1 and frame 2 combinations, and R-wave delay condition.

#### 2.2.3 Automatic cold pressor test

The timing, duration, and temperature of the bilateral feet cold pressor test (Larra et al., 2015, 2022) were controlled automatically using MATLAB. Water was preheated or cooled in two tanks located in an adjacent room. For the CPT condition, water temperature was maintained between 3–4°C, while for the control condition, it was kept at body temperature (36–37°C). Rectangular foot basins (35 × 20 × 19 cm) were filled and drained through a pipe and pump system, with each process taking less than 8 seconds. To prevent thermal stratification near the skin surface, the water in the basins was continuously circulated. Multiple sensors monitored the water level and temperature in real time, and cameras aimed at the basins ensured participants kept their feet submerged throughout the exposure. Both cold and warm water immersions lasted for 2 minutes. During this time, participants were not informed about the remaining duration of the exposure.

### 2.3 Data collection and analysis

#### 2.3.1 EEG

EEG recordings were obtained using a 64-channel active electrode cap (actiCAP; Brain Products GmbH, Munich, Germany) configured according to the extended 10/20 system, with a sampling rate of 1 kHz. FCz served as the online reference, and AFz as the ground. Electrode impedances were kept below 15 kΩ. Data acquisition was performed using a BRAINAMP DC amplifier (Brain Products GmbH, Munich, Germany).

Preprocessing and analysis of EEG data were conducted in MATLAB R2024b using the EEGLAB toolbox (v2024.2, Delorme & Makeig, 2004). First, boundaries in the EEG signal were removed after which they were filtered with a bandpass filter of 0.1–40 Hz (IIR Butterworth filter, 4th order, DC offset removed). Channels with flat signals were removed (*clean_flatlines*), and noisy channels were identified using a multi-criterion automatic rejection procedure based on statistical outlier detection. Specifically, channels were flagged for rejection if they exceeded a z-score threshold of 3.29 (Tabachnick & Fidell, 2007) on probability distribution, kurtosis, or spectral properties within the frequency range of 1–125 Hz. The union of channels identified by these three criteria was removed from the dataset (*pop_select*). On average, 6.78 (SD = 1.28, range = 3–9) channels were removed. Missing channels were then interpolated using spherical interpolation (*pop_interp*), followed by re-referencing to the average.

Artifact correction involved independent component analysis (ICA). Continuous data were first segmented into 4-second epochs time-locked to the onset of the first frame (from −1000 ms to +3000 ms). Trials containing artifacts were automatically excluded using an iterative rejection function (*pop_autorej*) with a voltage fluctuation threshold of 1000 µV, a probability threshold of 5 SD, and a maximum trial rejection rate of 5% per iteration. The remaining data were then downsampled to 250 Hz and filtered between 1 and 30 Hz (IIR Butterworth filter, 4th order).

ICA was performed using the Infomax algorithm with sub-Gaussian source detection enabled. The number of components was adjusted to the data rank using principal component analysis. Components were classified using the *ICLabel* algorithm (Pion-Tonachini et al., 2019) and those with less than 30% probability of reflecting brain activity were rejected. On average, 21.49 components (SD = 6.28, range = 5–36) were excluded. Refer to the Supplementary Materials (Supplementary Fig. S1) for a more detailed depiction of removed and retained ICs regarding the number, category, topographies and proportion of signal variance accounted for. The resulting ICA weights and component labels were transferred back to the original continuous dataset (1 kHz, bandpass filtered, interpolated, and average-referenced).

**Fig. 2.**
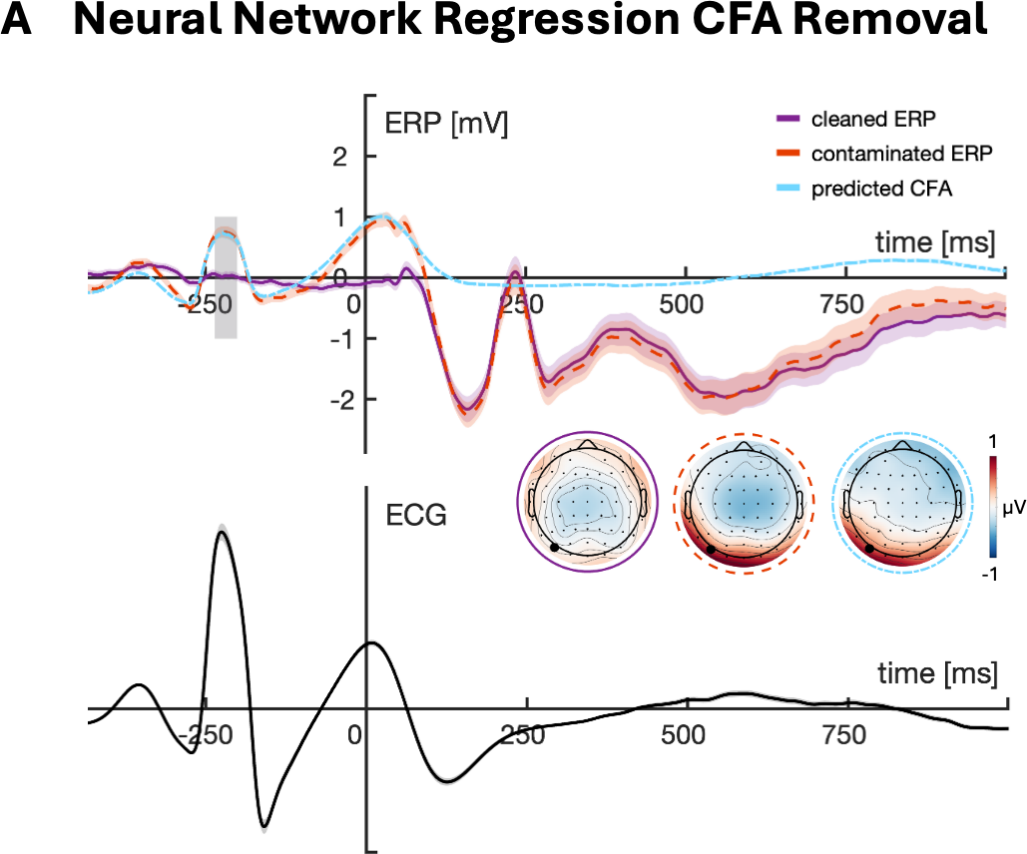
Removal of the cardiac field artefact via neural network regression using the *cfa_removal* toolbox (CFA, Arnau et al., 2023). Depicted is the Grand Average ERP at PO7 for the contaminated ERP, the predicted CFA, as well as the cleaned ERP. Shaded areas around lines represent 95% confidence intervals. Topographies are averaged over the R-Peak (vertical gray bar).

Cardiac field artifact (CFA) removal was performed in Python using the *cfa_removal* toolbox (https://github.com/stefanarnau/cfa_removal/tree/main), a nonlinear regression approach using neural networks to predict, and then subtract, the CFA from each EEG channel on a single‑trial basis (see Fig. 2). A detailed description of the method and technical parameters can be found in Arnau et al. (2023). In brief, the models are trained on stimulus-free EEG and ECG segments time‑locked to the R-peak of the ECG (−1000 ms to +1000 ms) to learn the mapping from ECG to CFA for each subject at each scalp electrode. The trained models are then used to generate trial-specific CFA estimates. These stimulus-free, predicted CFA waveforms are then subtracted from the EEG. Refer to the Supplementary Materials for examples of single subject plots used to visually confirm correct CFA removal (see Supplementary Fig. S1).

For analysis, the dataset was segmented into 2.5-second epochs relative to the change onset (−1000 to +1500 ms). Trials previously marked by *autorej* were rejected from this dataset, resulting in an average trial rejection rate of 9.68 % (SD = 4.27 percentage points). Analyses of event-related spectral perturbations (ERSP) were done on datasets filtered from 1–40 Hz, for ERPs signals were filtered at .01–40 Hz.

##### 2.3.1.1 Event-related lateralization (ERL)

We computed ERLs by subtracting contralateral minus ipsilateral ERPs for right and left targets to isolate lateralized processing from early to late stages at posterior sites (Coles, 1989; Wascher & Wauschkuhn, 1996). To investigate interaction effects of PHASE and CHANGE on early posterior ERLs, we applied the Factorial Mass Univariate ERP Toolbox (FMUT) from 0–400 ms after change onset on a posterior region: ‘PO3/PO4’, ‘PO7/PO8’, ‘PO9/PO10’, ‘P1/P2’, ‘P3/P4’, ‘P5/P6’, ‘P7/P8’, ‘O1/O2’ (Fields & Kuperberg, 2020). FMUT enables mass univariate ANOVA analyses within factorial designs, using cluster-based permutation testing to control for multiple comparisons. Because FMUT does not support unbalanced group designs, we conducted additional direct group comparisons between groups using the *clustGRP* function from the Mass Univariate ERP Toolbox (Groppe et al., 2011), which is based on cluster-level t-statistics. For both FClustGND and clustGRP, we ran 10,000 permutations to derive *p*-values using a spatial–temporal clustering criterion of 39 and an alpha level of .05 and the default two-tailed setting (tail = 0), which conducts separate upper- and lower-tailed tests and applies a Bonferroni correction.

We used a method described by Ülkü et al. (2024, 2025) to quantify the contribution of individual channels using a normalized contribution metric. This metric was calculated as the proportion of significant datapoints per channel relative to the channel with the highest number of significant points within the cluster (threshold 70%).

##### 2.3.1.2 Residue iteration decomposition (RIDE)

To dissociate between stimulus and response related ERL effects, Residue Iteration Decomposition (RIDE) was applied to the EEG data. RIDE allows to account for latency variability in event-related potentials (ERPs) by decomposing ERP signals into distinct component clusters based on their temporal alignment with specific events (Ouyang et al., 2015a, 2015b). Specifically, RIDE separates EEG activity into three components: the stimulus-locked component (S-cluster), the response-locked component (R-cluster), and a component that varies in latency across trials but is not strictly time-locked to either stimulus or response (C-cluster). The S-cluster captures activity tightly locked to stimulus onset and is estimated by aligning and averaging the EEG data to stimulus markers, in our case the change onset. The R-cluster is derived from data time-locked to the participant’s response. The C-cluster is estimated iteratively by removing the contributions of the S- and R-clusters and aligning the residual activity to its estimated peak latencies. The decomposition was performed using the RIDE toolbox on change-locked epochs in MATLAB following the recommended pipeline and default parameters of *RIDE_call* (Ouyang et al., 2015a, 2015b).

To investigate interaction effects of PHASE, CHANGE, and GROUP on the RIDE response cluster, we again used *FClustGND* and *clustGRP* as described above, however including all homologues channel pairs in a time range from 0 to 900 ms.

##### 2.3.1.3 ERSPs

To analyze ERSPs, we extracted frequency components between 4 and 30 Hz in logarithmic steps via wavelet convolution, applied separately to each channel. The wavelet’s full width at half-maximum ranged from 750 ms (low frequencies) to 100 ms (high frequencies). We applied baseline correction using the time window from −200 ms to 0 ms relative to the change-onset.^1^

To assess whether processing of spatial conflicts was altered by differences in conflict monitoring and cognitive control, we analyzed midfrontal theta-band (4–8 Hz) ERSPs at a midfrontal region of interest (channels: Fz, F1, F2, FCz, FC1, FC2, Cz, C1, C2) (Asanowicz et al., 2021; Cohen, 2014; Muralidharan et al., 2023; Tollner et al., 2017).

To analyze change-related contra-ipsilateral modulations of alpha power, we calculated the LPS index (Van der Lubbe & Utzerath, 2013). The LPS index is computed similarly to the double subtraction technique in ERLs. Specifically, we subtracted contra- from ipsilateral alpha power at the homologue electrode pair PO7 and PO8 separately for left and right targets and scaled their average by the summed activation of both hemispheres.

Finally, we conducted cluster-based permutation tests using *ft_freqstatistics* (estimation method: Monte-Carlo, cluster correction: .05, cluster statistic: max t-sum, permutations: 10,000, two-sided) from the FieldTrip toolbox (Oostenveld et al., 2011) to evaluate the effects of change, phase, and group on midfrontal theta and LPS.

#### 2.3.2 Cardiovascular measures

Heart rate (HR) was continuously recorded using three electrodes in a modified Lead II chest configuration, connected to an AccuSync 72 ECG monitor (AccuSync Medical Research Corporation; Connecticut, Milford, USA). Offline artifact correction and R-peak detection were performed using *RDECO* (Moeyersons et al., 2019), with subsequent visual inspection to ensure accurate detection. IBIs containing extrasystoles were excluded from analysis. For T-wave peak and offset detection, we applied the *ecg_process* and *ecg_delineate* functions from *NeuroKit2* (Makowski et al., 2021), which were used to calculate the cardiac angle at change onsets. We calculated RMSSD using *CreateTimeAnalysis* from *RHRV* (Rodriguez-Linares et al., 2008). To adjust to small deviations in block length between participants which would sometimes result in different number of analysis bins whereby the last bin would contain less actual data (e.g., 14-minute block/2-minute bins = 7 blocks versus 15/2 = 7.5 with the last 2-minute bin only containing 30 seconds of actual data), instead of fixed bins we calculated normalized analyses bins, that is, 6 analysis bins for experimental blocks which allowed us to retain all data and comparability between bins. Two participants were excluded from the manipulation check HR and HRV analysis due to missing data in one analysis bin.

Blood pressure was measured using a remote controlled Dinamap Pro 300 V2 (Critikon; Tampa, Florida, USA). Baseline blood pressure readings were taken at 3-minute intervals during the post-training resting phase. During the Cold Pressor Test (CPT) and the control condition, blood pressure was measured 1 minute after water immersion. Due to technical issues with the Dinamap blood pressure readings were unavailable for eight participants.

#### 2.3.3 Behavioral data

Response times (RT) quicker than 150 ms and slower than 1000 ms were excluded from RT analysis (cf. Supplementary Table S1). Analysis of RTs was performed on accurate trials only. Trials with wrong responses were considered errors, that is, errors of commission, whereas trials with RTs higher than 1000 ms were considered misses, that is, errors of omission. In case of orientation changes (ORI), trials were considered correct when no response was executed.

#### 2.3.4 ANOVA

Statistical analysis was performed using R 4.3.1 (R Core Team, 2023) and RStudio 2025.05.0 + 496 “Mariposa Orchid” Release (Posit team, 2025).

To account for the unbalanced group sizes, we conducted robust repeated-measures ANOVAs using the *RM* function from the MANOVA.RM R package (Friedrich et al., 2019). This function uses parametric bootstrapping to obtain *p*-values for the Wald-type statistic (WTS), ensuring robustness against violations of normality and homogeneity of variance (10,000 permutations). For confirmatory follow-up analyses, we employed linear mixed-effects models using the *lmer* function from the lme4 package (Bates et al., 2015). Estimated marginal means (EMMs) were calculated using the *emmeans* package (Lenth, 2025) to examine main and interaction effects. Post-hoc comparisons of EMMs were adjusted using the Benjamini-Hochberg procedure. Effect sizes were computed using *t_to_d* from the *effectsize* package (Ben-Shachar et al., 2020). Bayes factors were computed using the *ttestBF* from the BayesFactor package (Morey & Rouder, 2012).

For the behavioral measures, we ran separate 2 × 2 × 4 robust repeated-measures ANOVAs with the between factor GROUP (CPT vs. Control) and within factors PHASE (Systole vs. Diastole) and CHANGE (LOB vs. LOU vs. LUM vs. ORI). For RTs and misses the factor CHANGE did not include the ORI level as it was a no-go condition.

For the analysis of HR, MAP, and RMSSD, the ANOVAs included the between factor GROUP (CPT vs. control) and the within factor TIME representing the different experimental phases (i.e., 9 bins: resting phase, 4 CPTs, 4 Blocks).

#### 2.3.5 Regression

To probe the previously reported cardiovascular correlates of cardiac cycle effects, we conducted regression analyses on participants in the CPT group using both a robust frequentist and a Bayesian model. We probed previously suggested drivers of cardiac cycle effects, specifically SBP during experimental blocks as a proxy for baroreceptor signal strength during systole (c.f. Lacey & Lacey, 1978; Leupin & Britz, 2024), RMSSD during experimental blocks as a measure of HRV (c.f. Al et al., 2020), and SBP and HR responses to CPT (CPT – Resting) as indicators of homeostatic regulatory function (c.f. Hines & Brown, 1936; Lovallo, 1975). Also, to control for differences in HR response due to underlying HR differences, we added the interaction with HR measured during experimental blocks (Δ Phase Effect (Systole-Diastole) ~ RMSSD * SBP * Δ SBP * Δ HR + Δ HR * HR).

Prior to modeling, extreme multivariate outliers were identified and removed based on Mahalanobis distance using *mv_outlier()* from the *MVN* package (Korkmaz et al., 2013). We ran these regression models on the behavioral (LOB errors, LUM misses) as well as EEG effects (LOB RIDE, LUM RIDE, LOB Theta). Predictors and outcome measures were z-standardized before analysis. Missing data were handled by listwise deletion. Final sample size for the models was N = 42.

For the robust frequentist model, we fit an MM-estimator using *lmrob()* from *robustbase* (setting = “KS2014”, nResample = 100000, max.it = 1e5, rel.tol = 1e-7, refine.tol = 1e-7, method = “MM”; Maechler et al., 2006). Marginal effects were estimated with *predict_response()* from the *ggeffects* package (Lüdecke, 2017) and interaction probing used the Johnson–Neyman procedure via *johnson_neyman()* from the *interactions* package (Long, 2019). For the Bayesian analysis we fit the identical models using *brms* (Bürkner, 2015) with a Student-t observation model, weakly informative priors on slopes and intercept, and sampling parameters chains = 4, iter = 10000, warmup = 1000. Posterior summaries and checks were done with *describe_posterior()* from *bayestestR* (Makowski, Lüdecke, et al., 2019) and *bayes_R2()* from *brms*.

## 3 Results

### 3.1 Manipulation check

**Fig. 3.**
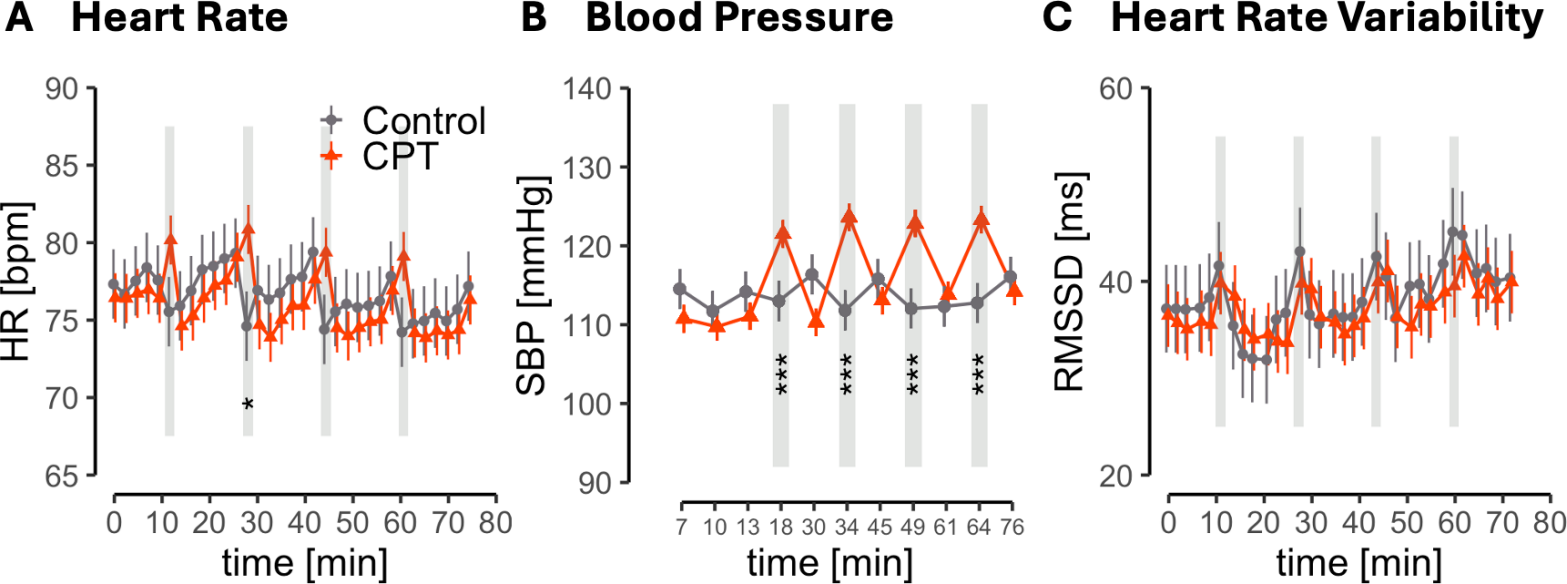
Heart rate (A), systolic blood pressure (B) and RMSSD (C) during the experiment for CPT and control group. Gray bars mark the timing of CPT or warm water control procedure. Error bars show standard errors. *p < .05, ***p < .001.

To verify the efficacy of CPT exposure, we examined heart rate, systolic blood pressure, and heart rate variability (HRV) across the experimental time course (Fig. 3). To enhance readability, only the central results are reported here. Find detailed statistics in the Supplementary Materials.

Analyses showed a significant effect of TIME (WTS_8_ = 69.29, *p* < .001) and a TIME × GROUP interaction (WTS_8_ = 44.07, *p* < .001) on heart rate (see Fig. 3A). Simple effect analyses revealed that while heart rate of CPT and control group did not differ at baseline (resting phase: *t*(73) = -0.32, *p* = .750, *d* = 1.04, BF_10_ = 0.26), heart rate in the CPT group was significantly increased during the second CPT (*t*(73) = 2.06, *p* = .043, *d_z_* = -6.69, BF_10_ = 1.5). No other time-point contrast or the main effect of GROUP (WTS < 1) was significant (see Supplementary Table S2).

As displayed in Figure 3B, SBP was significantly influenced by exposure to the CPT as indicated by a significant effect of TIME (WTS_9_ = 80.9, *p* < .001) and GROUP*TIME (WTS_9_ = 104.74, *p* < .001). Simple effect analyses showed that CPT and control group did not differ at baseline during the resting phase (*t*(67) = 1.14, *p* = .260, *d* = 0.28, BF_10_ = 0.45) but during all CPTs SBP was significantly increased in the CPT group (all *p* ≤ .012). Groups did not significantly differ at other timepoints or overall (GROUP: WTS_1_ < 1; see Supplementary Table S3).

The robust ANOVA on RMSSD showed a significant main effect of TIME (WTS_8_ = 60.42, *p* < .001) but not GROUP (WTS_8_ < 1) or GROUP*TIME (WTS_8_ = 3.48, *p* = .928) as displayed in Figure 3C. Refer to the Supplementary Materials for the detailed TIME results (see Supplementary Table S4).

### 3.2 Behavioral measures

#### 3.2.1 Change effects

**Fig. 4.**
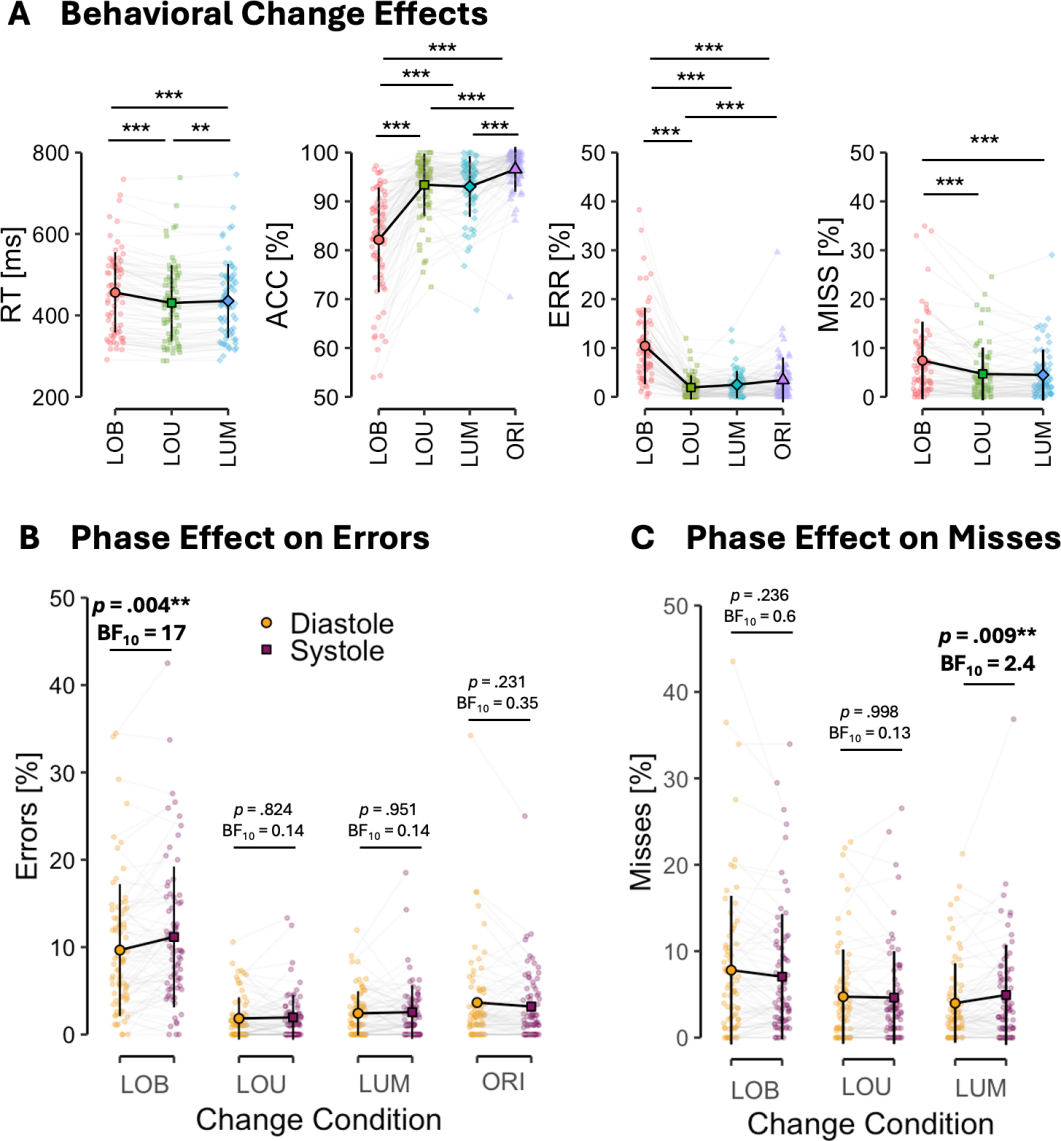
Effects of different change conditions on behavioral measures reaction times, accuracy, errors, and misses (A). Interaction effects of change and phase effects for errors (B), and misses (C). Error bars show standard deviation. **p < .01, ***p < .001.

As displayed in Figure 4A, the change detection task produced the expected behavioral effects as indicated by significant main effects of CHANGE on RTs (WTS_2_ = 114.367, *p* < .001), accuracy (WTS_3_ = 166.451, *p* < .001), errors (WTS_3_ = 126.952, *p* < .001), as well as misses (WTS_2_ = 36.308, *p* < .001) indicating decreased performance following perceptual conflicts in LOB trials, as well as facilitated performance depending on target salience. To enhance readability, we report detailed statistical on change effects in the Supplementary Materials (Change Effects section).

#### 3.2.2 Phase effects

Robust ANOVAs revealed a significant interaction effect of CHANGE*PHASE on errors (WTS_3_ = 10.34, *p* = .027) as well as misses (WTS_1_ = 8.16, *p* = .022) (see Fig. 4B).

Simple effect analyses showed an LOB-specific effect of PHASE on errors (WTS_1_ = 8.16, *p* = .007) indicating increased error during systole versus diastole in conflict trials (*t*(74) = -2.99, *p* = .004, *d_z_* = -0.35, BF_10_ = 17). There was no effect of PHASE in LOU (WTS_1_ = 0.05, *p* = .817, *t*(74) = -0.22, *p* = .824, *d_z_* = -0.03, BF_10_ = 0.14), LUM (WTS_1_ = 0.004, *p* = .94, *t*(74) = -0.06, *p* = .951, *d_z_* = -0.01, BF_10_ = 0.14), and ORI (WTS_1_ = 1.65, *p* = .201, *t*(74) = 1.26, *p* = .213, *dz* = 0.15, BF_10_ = 0.35) changes.

In misses, there was an LUM specific effect of PHASE (WTS_1_ = 5.84, *p* = .021) showing increased misses of LUM changes during systole versus diastole (*t*(74) = -2.70, *p* = .009, *d_z_* = -0.31, BF_10_ = 2.4). Phase did not affect misses in LOB (WTS_1_ = 1.42, *p* = .237, *t*(74) = 1.20, *p* = .236, *d_z_* = 0.14, BF_10_ = 0.6) and LOU changes (WTS_1_ = 0, *p* = .998, *t*(74) = 0.00, *p* = .997, *d_z_* = 0.00, BF_10_ = 0.13).

There were no further significant main or interaction effects on RT (GROUP: WTS_1_ = 2.603, *p* = .117; PHASE: WTS_1_ = 1.422, *p* = .237, GROUP*PHASE: WTS_1_ = 3.841, *p* = .055; PHASE*CHANGE: WTS_1_ = 2.35, *p* = .325; all other WTS < 1), accuracy (PHASE: WTS_1_ = 3.123, *p* = .084; GROUP*CHANGE: WTS_1_ = 2.345, *p* = .526; PHASE*CHANGE: WTS_1_ = 8.665, *p* = .053; all other WTS < 1), errors (GROUP: WTS_1_ = 1.35, *p* = .249; PHASE: WTS_1_ = 2.614, *p* = .113; GROUP*CHANGE: WTS_3_ = 2.639, *p* = .471; all other WTS < 1), and misses (GROUP: WTS_1_ = 1.717, *p* = .2; GROUP*PHASE: WTS_1_ = 3.94, *p* = .057; GROUP*CHANGE: WTS_2_ = 1.24, *p* = .552; all other WTS < 1).

### 3.3 Posterior ERLs

Results of the cluster-based permutation statistic at the posterior and parietal-occipital region of interest showed that the visual changes evoked significant lateralization in the time range of the change positivity and the contralateral posterior negativity components and confirmed successful salience manipulation of the change conditions. However, they did not show main or interaction effects of GROUP or PHASE (c.f. Supplementary Materials, Table S5).

**Fig. 5.**
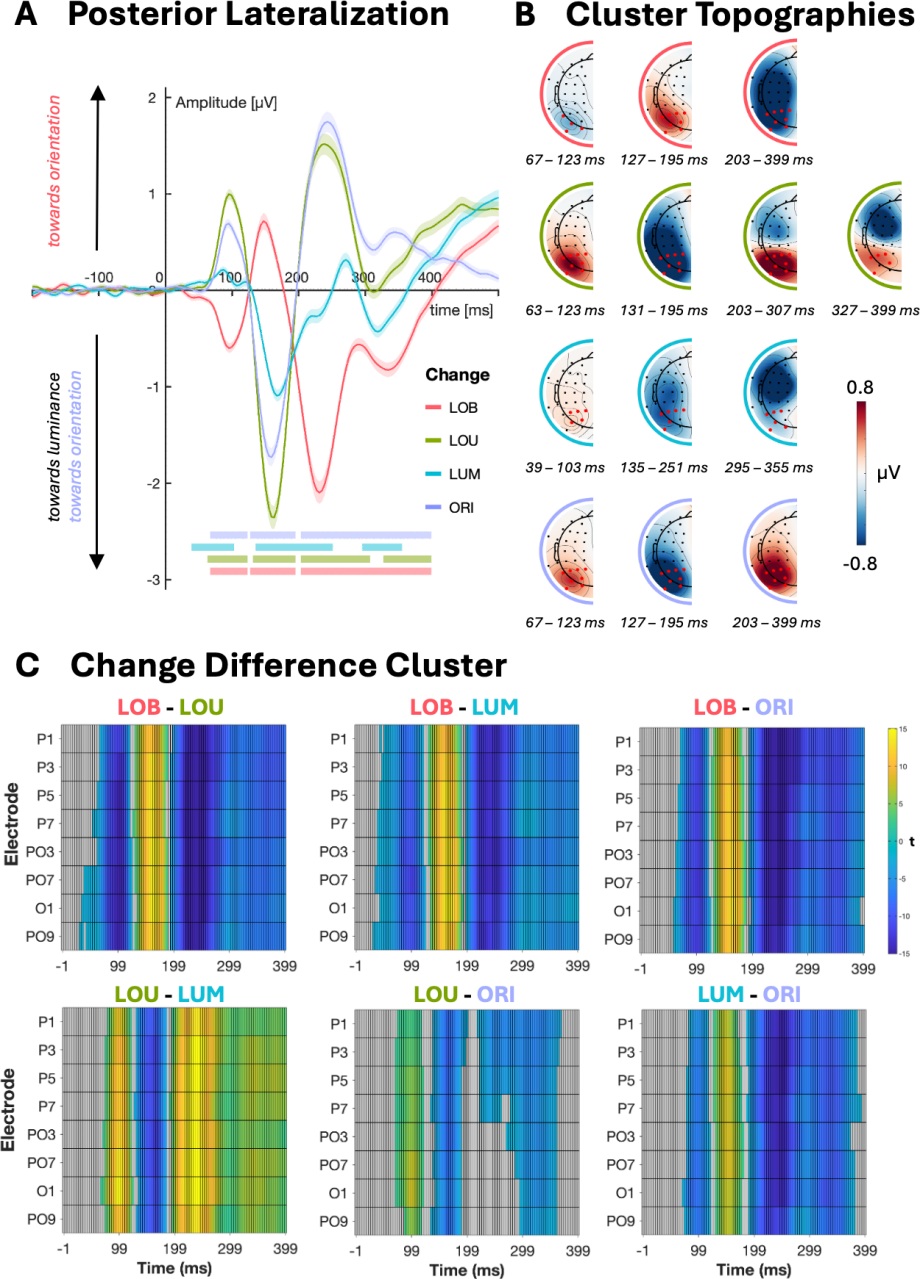
Event-related lateralization (ERL) averaged over posterior region of interest (PO3/PO4, PO7/PO8, PO9/PO10, P1/P2, P3/P4, P5/P6, P7/P8, O1/O2) by change condition (A). To enable easier comparison between change conditions, the sign of ERLs for ORI changes was flipped, that is, “towards target” corresponds to the location of the orientation change. Shaded areas around ERLs represent 95% confidence intervals. Horizontal bars show clusters indicating significant lateralization. Topographies show corresponding clusters where channels higher 80% significant timepoints are highlighted in red (B). Results of the cluster-based permutation test on differences between change conditions whereby color indicates significant differences between change conditions (C).

As displayed in Figure 5, LOB trials showed significant negative lateralization from 67–123 ms at posterior and parietal-occipital sites indicating a higher change positivity toward the more salient orientation change (*p* = .005, *M* = -756.56). Furthermore, there was a significant positive lateralization in the N1pc range (127–195 ms, *p* = .017, *M* = 395.42) indicating initial orientation towards the orientation change versus the luminance change. Finally, a later cluster showed more sustained posterior lateralization in the time range of the N2pc and N3pc components (203–399 ms, *p* < .001, *M* = -3470.1).

In contrast to the LOB condition, the other change conditions displayed a positive lateralization in the time range of the change positivity, that is, LOU trials from 63–123 ms (*p* < .001, *M* = 1072.4), LUM trials from 39–103 ms (*p* = .009, *M* = 330.1), and ORI trials from 67–123 ms (*p* = .002, *M* = 784.2). As shown in Figure 5C, LOU trials produced stronger change positivity compared to LUM (*p* < .001, *M* = 789.17) and ORI trials (*p* = .003, *M* = 386.25) confirming higher salience of the combined luminance-orientation change versus unilateral luminance or orientation changes. Comparison of the LUM and ORI condition changes showed increased lateralization for unilateral orientation changes versus luminance changes (*p* = .016, *M* = -443.58).

In the time range of the N1pc, there was negative lateralization for LOU (*M* = -1313, *p* < .001) and LUM (*M* = -1549, *p* < .001) changes confirming visual orientation toward the luminance respectively the orientation changes in ORI trials (*p* < .001, *M* = -1325). Again, lateralization increased depending on the salience of the target, that is, LOU changes showed an increased N1pc versus LUM (*M* = -859.75, *p* < .001) and ORI changes (*M* = -586.09, *p* < .001) whereby unilateral orientation changes produced higher lateralization versus unilateral luminance changes (LUM-ORI: *M* = -556.84, *p* = .01).

Finally, multiple clusters indicated later lateralized components following LOU, LUM, and ORI changes. In LOU trials, cluster‐based permutation revealed a robust positive lateralization in the N2pc time window (*M* = 1420, *p* < .001), spanning approximately the 200–310 ms interval. The same component was visible following ORI changes, forming a larger significant cluster (*M* = 3134, *p* < .001) that extended roughly from 200 ms to 400 ms. LUM changes produced significant negative lateralization approximately spanning from 295–355 ms (*M* = -366.5, *p* = .007), that is, in the N3pc range indicating later reorientation toward the target location. Finally, there was a late positive lateralization following LOU changes ranging from approximately 327–399 ms (*M* = -366.5, *p* = .007).

### 3.4 Response-cluster (RIDE)

**Fig. 6.**
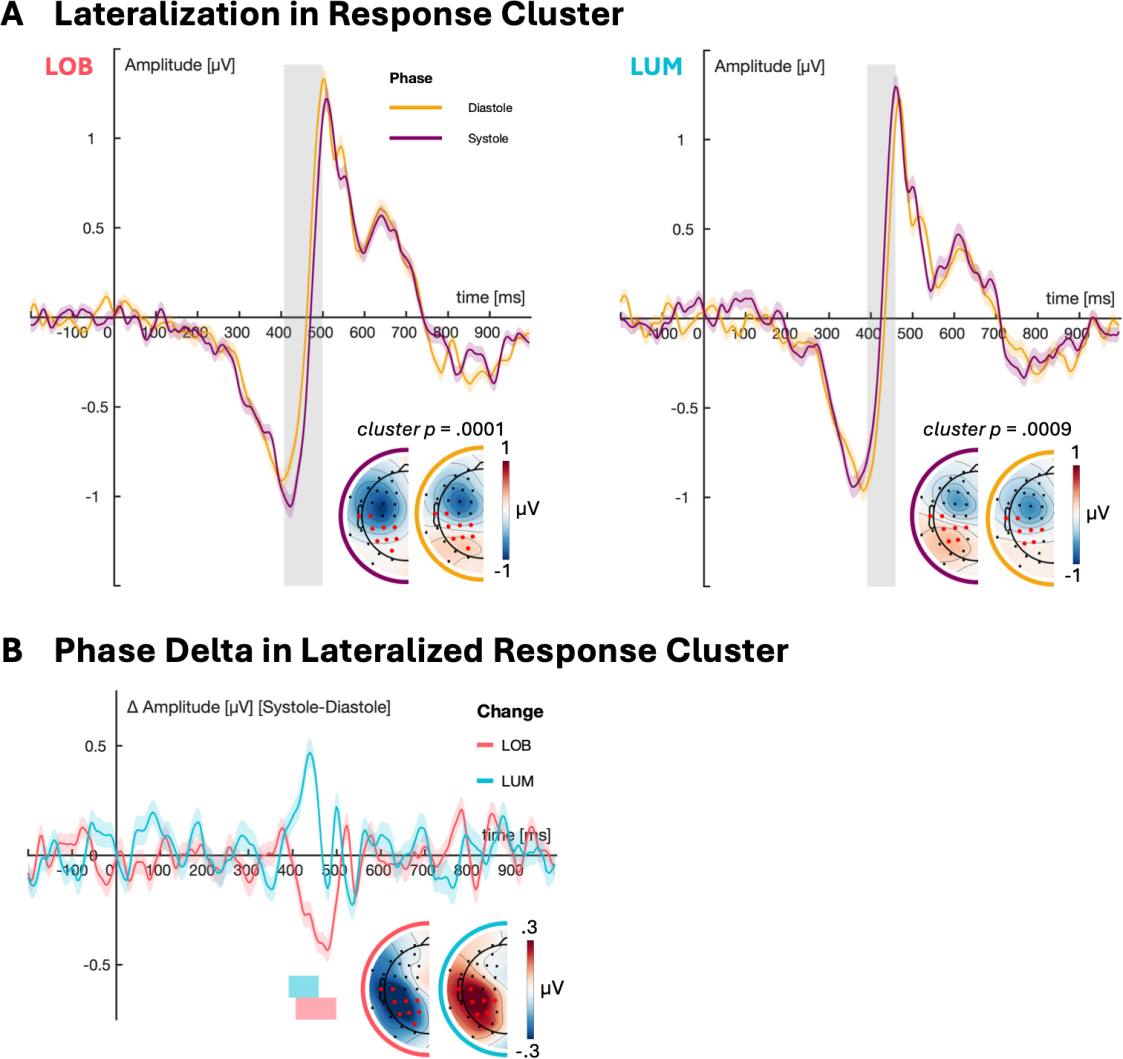
Significant phase effects in the RIDE response cluster for LOB and LUM changes (A). Difference Potentials (Systole-Diastole) by cluster (B). Shaded areas around ERLs represent 95% confidence intervals. Shaded bars indicate significant differences. Topographies show mean amplitudes at significant timepoints. Channels that contribute most to a cluster (i.e., have a minimum of 70% of all significant timepoints) are highlighted in red.

Following up our behavioral effects, cluster-based permutation analyses on PHASE*CHANGE interactions on the RIDE response-locked cluster revealed phase effects on response related lateralization. As displayed in Figure 6, there was a significant interaction of PHASE*CHANGE (*M* = 4566.34, *p* = .0001). Follow-up phase contrasts showed LOB und LUM-specific PHASE effects. For LOB changes, response-locked lateralization at centroparietal sites was significantly increased during systole versus diastole from 407 to 499 ms (*F_max_* = 5652.73, *p* = .0002, spatial mass peak: CP3, temporal mass peak: 459 ms). The opposite was the case for LUM changes where lateralization was significantly smaller during systole compared to diastole from 391 to 459 ms (*F_max_* = 3215.36, *p* = .0009, spatial mass peak: CP3, temporal mass peak: 439 ms). CPT and control group did not differ significantly in LOB systole (*p_min_* = 1.036) and diastole (*p_min_* = 0.081), as well as LUM systole (*p_min_* = 0.3262) and diastole trials (*p_min_* = 0.2974).

### 3.5 ERSPs

#### 3.5.1 Midfrontal theta

**Fig. 7.**
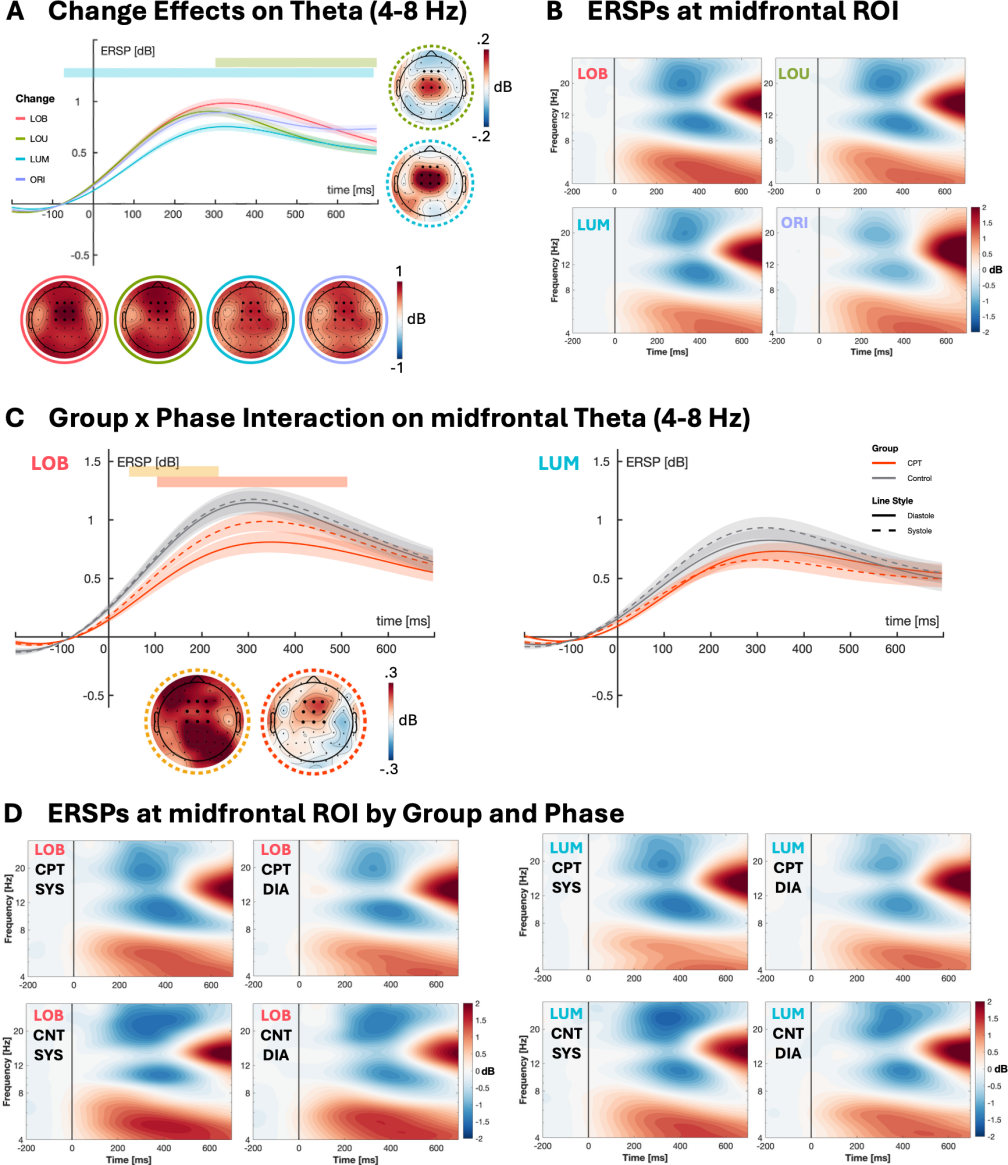
Midfrontal theta (4–8 Hz) by change condition with colored bars showing significant differences of LOB versus LOU and LUM changes (A). Topographies with dotted outlines show significant differences in theta power and topographies with solid outlines averaged theta power ± 20 ms around peaks (solid outlines). Time-frequency maps of change conditions at the same midfrontal region of interest (B). Midfrontal theta power by group and phase for LOB and LUM changes (C) with colored bars and topographies showing significant differences, that is, CPT versus Control in diastole trials as well as systole versus diastole in CPT group. Time-frequency maps for midfrontal electrodes by group and phase for LOB and LUM changes (D). Shaded areas around lines represent 95% confidence intervals. Horizontal bars indicate significant clusters.

As displayed in Figure 7A, midfrontal theta showed the expected effects of CHANGE, that is, LOB changes showed higher midfrontal theta versus LOU (*M* = 350.43, *p* = .005, significant timepoints: 300–696 ms) and LUM (*M* = 880.01, *p* < .001, significant timepoints: -72–688 ms), but not ORI changes (*M* = 40.62, *p* = .092).

Based on the behavioral effects, contrasting systole and diastole onsets for LOB and LUM changes showed that midfrontal theta was significantly lower during systole specifically for LOB trials (*M* = 226.23, *p *= .014, significant timepoints: 128–508 ms). As displayed in Figure 7C, this effect was dependent on GROUP, that is, only in the CPT group systole trials showed higher midfrontal theta (*M* = 259.32, *p* = .011, significant timepoints = 104–512 ms). Contrasting the groups showed that midfrontal theta was lower in the CPT group specifically in diastole trials (*M* = 99.49, *p* = .04, significant timepoints = 44–236 ms), while there was no significant difference between groups in systole trials. There were no significant main or interaction effects of phase or group on midfrontal theta following LUM changes.

#### 3.5.2 LPS

**Fig. 8.**
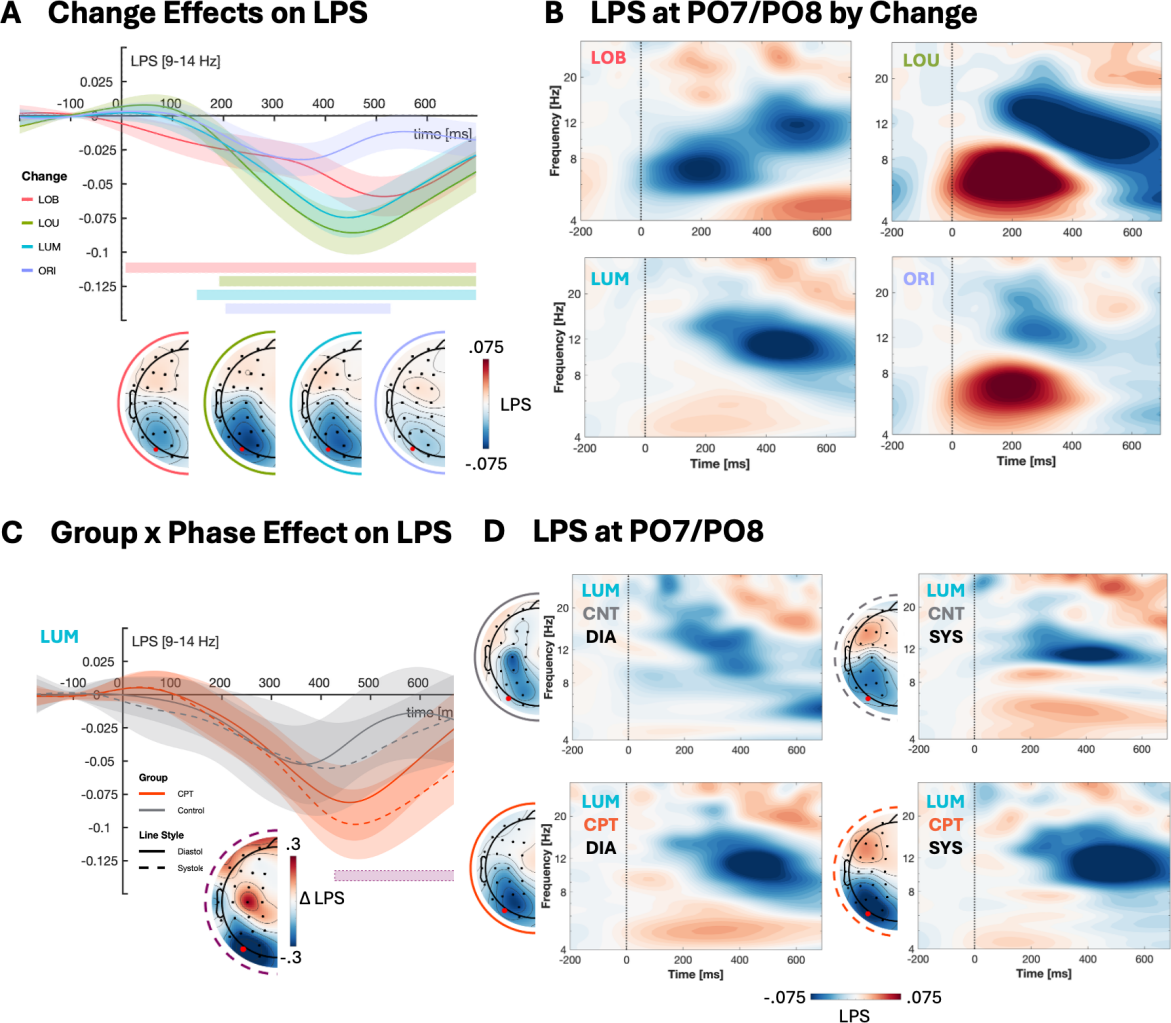
LPS (9–14 Hz) at PO7/PO8 by change condition with colored bars indicating significant lateralization and corresponding topographies (A). Time-frequency maps of change conditions at PO7/PO8 (B). Interaction effect of GROUP and PHASE following LUM changes with purple bar indicating significantly different timepoints between CPT and control group in systole trials (C). Time-frequency maps showing LPS for LUM changes by GROUP and PHASE at PO7/PO8 (D) and corresponding topographies averaged over the significant cluster timepoints and the frequency range of interest (9–14 Hz). Shaded areas around lines represent 95% confidence intervals. Horizontal bars indicate significant clusters. Negative LPS indicates that the contralateral alpha power was reduced compared to the ipsilateral power.

As displayed in Figure 8, we found significant lateralization in posterior alpha power in all change conditions. Specifically, we found negative LPS toward the luminance change following LOB (*M* = -810.06, *p* < .001, significant timepoints = 8–696 ms), LOU (*M* = -851.08, *p* < .001, significant timepoints = 192–696 ms), and LUM (*M* = -847.95, *p* < .001, significant timepoints = 148–696 ms) changes indicating lower contra- versus ipsilateral alpha power. Analogue, following ORI changes, there was significant lateralization toward the orientation change (*M* = -310.14, *p* = .001, significant timepoints = 204–528 ms).

Additionally, we found a significant group difference for LUM changes specifically for systole onsets. Specifically, the CPT group showed significantly increased LPS compared to the control group (M = 58.18, *p* = .017, significant timepoints = 428–672 ms). There were no further main or interaction effects of group and phase.

### 3.6 Cardiovascular drivers

To probe cardiovascular drivers of the observed cardiac cycle effects, we modeled the behavioral and EEG effects on participants of the CPT group (following multivariate outlier rejection, N = 43) using a robust regression with MM-estimators and a confirmatory Bayesian regression with a Student-t likelihood. Specifically, we used SBP during experimental blocks as a proxy for baroreceptor signal strength during systole (c.f. Lacey & Lacey, 1978; Leupin & Britz, 2024), RMSSD during experimental blocks as a measure of HRV (c.f. Al et al., 2020), and SBP and heart rate responses to CPT as indicators of homeostatic regulatory function (c.f. Hines & Brown, 1936; Lovallo, 1975).

**Fig. 9.**
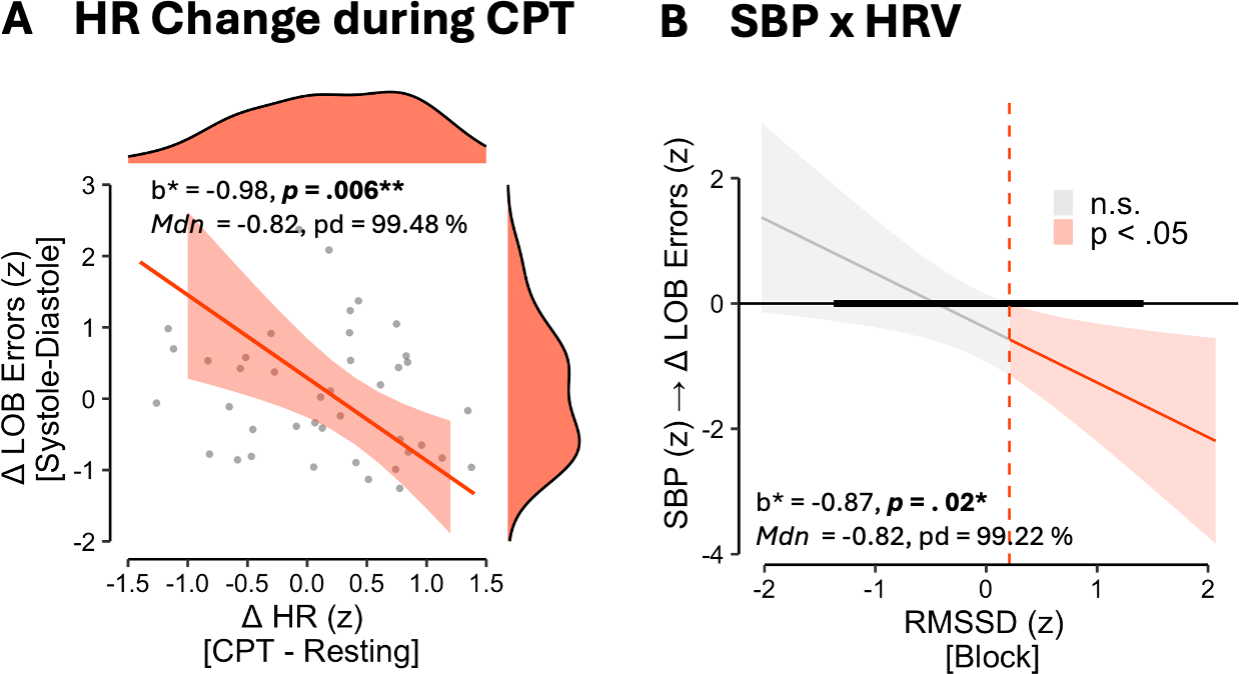
Significant cardiovascular predictors of the phase difference in LOB errors. (A) Shows association of heart rate (HR) response to the CPT. (B) Shows Johnon-Neyman plot of the interaction of systolic blood pressure (SBP) and heart rate variability (HRV, RMSSD) whereby the thicker horizontal line indicates the data range of RMSSD (z). Shaded areas around represent 95% confidence intervals. *p < .05, **p < .01.

The model on LOB errors showed two significant effects for the phase difference in LOB errors (R² = .582, RSE = .646; Bayesian R² = .528, 95% CI [.381, .628]). As displayed in Figure 9A, HR response to the CPT showed a negative association indicating that participants with a decrease in heart rate showed higher difference in LOB errors between systole and diastole (b* = -0.98, SE = 0.32, *p* = .006; *Mdn* = -0.82, 90% CI [-1.31, -0.31], pd = 99.48 %, ROPE ≈ 0%).

Second, the models revealed that RMSSD moderated the relationship of SBP and Phase Errors (b* = -0.87, SE = 0.35, *p* = .02; *Mdn* = -0.82, 90% CI [-1.35, -0.28], pd = 99.22 %, ROPE ≈ 0%). Johnson-Neyman plots indicated that at increased RMSSD, SBP is negatively associated with the phase difference in LOB errors (*p* < .05 when RMSSD outside [-2.80, 0.21], RMSSD data range = [-1.34, 1.39]).

There were no further significant main or interaction effects on LOB errors, LUM misses, and the phase difference in the respective RIDE response cluster respectively on the LOB Theta effects (see Supplementary Materials, Tables S6–S10). Variance inflation factors indicated no problematic multicollinearity in any of the models. Bayesian model convergence was good for all models (Rhat = 1.000, ESS ≥ 18437.00). Posterior predictive checks confirmed an adequate fit to the data.

## 5 Discussion

This study investigated how variations in cardioafferent signaling modulate visual change detection and spatial conflict processing. Visual changes were presented during either ventricular systole or diastole, corresponding to phases of high versus low baroreceptor activity. To probe cardiovascular correlates of the observed effects, participants were assigned to either a control or CPT group, the latter undergoing an automatized feet cold pressor test (CPT) before each experimental block reliably elevating systolic blood pressure (Bachmann et al., 2018; Larra et al., 2015, 2020; von Haugwitz et al., 2024).

The change detection task manipulated target salience and introduced spatial conflicts by pairing task relevant unilateral luminance changes in two symmetrical lateral bars (LUM) with a more salient orientation change either at the same location (LOU) or in the opposite hemifield (LOB), while isolated orientation changes served as catch trials (ORI). LOU changes yielded faster and more accurate responses relative to LUM, whereas LOB changes slowed responses and increased errors, replicating the expected effects of this task (Fig. 4A; Labrenz et al., 2012; Schneider et al., 2012b; Wascher & Beste, 2010; Wascher et al., 2012).

EEG analyses further supported these behavioral findings (Fig. 5). LUM changes elicited posterior lateralization’s in the change positivity and N1pc time ranges, which were amplified for LOU changes, indicating enhanced change detection and early attentional orienting (Busch et al., 2010; Schneider & Wascher, 2013; Schneider et al., 2012a; Verleger et al., 2012; Wascher & Beste, 2010). In LOB trials, these early components were biased towards the orientation change, followed by later N2pc/N3pc shifts toward the luminance change, reflecting additional target selection and distractor suppression, and confirming occurrence of spatial conflicts (Eimer, 1996; Hickey et al., 2009; Luck & Hillyard, 1994; Verleger et al., 2012; Wascher & Beste, 2010; Woodman & Luck, 1999). In line with this, LOB trials also produced stronger midfrontal theta synchronization compared to LOU and LUM changes, consistent with heightened cognitive control demands and conflict processing (Asanowicz et al., 2021; Cohen, 2014; Muralidharan et al., 2023; Tollner et al., 2017). Finally, ORI trials elicited larger change positivity and N1pc amplitudes compared to LUM changes, confirming higher salience of orientation versus luminance changes.

Cardiac cycle phase modulated performance selectively in LUM and LOB trials (Fig. 4). In LOB trials, participants committed more errors when changes occurred during systole, tending to respond to the location of the salient orientation rather than to the task-relevant luminance change. In LUM trials, misses were more frequent for systolic compared to diastolic onsets. No cardiac phase effects emerged for LOU or ORI trials, in neither errors nor misses. This pattern highlights several aspects of the specificity of the LOB and LUM effects. First, the findings cannot be explained by a general suppression of perceptual processing of visual stimuli during systole (e.g., McIntyre et al., 2008; Stewart et al., 2006; Walker & Sandman, 1982). Second, they cannot be attributed solely to an increased response tendency toward salient orientation changes, as systolic onsets did not modulate accuracy in ORI trials. Finally, while LUM changes were more likely to be missed during systole, such misses do not appear to underlie the effect in LOB trials. If this were the case, LOB changes would be processed similarly to ORI changes and should therefore produce more misses; instead, they elicit more errors, indicating that participants responded but directed their responses toward the orientation rather than the luminance change. Taken together, these results suggest that presenting changes during systole specifically disrupts the processing of spatial conflicts introduced by salient distractors (as in LOB trials), while also increasing the likelihood of missing less salient luminance changes (as in LUM trials) when presented in isolation.

While theoretically attributable to attentional-perceptual inhibition, our EEG results suggest that these behavioral effects emerged not during early stimulus processing but rather in later response-related activity, specifically during premotor response encoding (Fig. 6). Contrary to our hypothesis, we did not find modulations of early stimulus-locked posterior ERL, that is, no difference emerged between systole and diastole in the time range of the change positivity, or contralateral posterior negativities. Rather, we found that cardiac cycle phase affected lateralization in the response cluster, as confirmed by the RIDE analysis. Specifically, in LOB trials, systolic onsets evoked significantly greater central–posterior lateralization compared to diastolic onsets from approximately 407 to 499 ms with the cluster spatial mass peaking at CP3. In LUM trials, this relationship was inverted, with changes during systole evoking reduced lateralization relative to diastole from approximately 391 to 459 ms with the spatial mass again peaking at CP3. Previous studies have identified central–posterior lateralization in this time and electrode range as direction encoding lateralization (DEL, Wascher et al., 1999; Willemssen et al., 2004). DEL emerges over central–parietal scalp sites and indexes premotor response encoding via a visuo‐motor pathway which precedes and is topographically distinct from the classical motoric lateralized readiness potential at frontocentral sites. Crucially, DEL amplitude has been shown to vary with stimulus-response compatibility, increasing in incompatible compared to compatible trials (Willemssen et al., 2004).

Such altered or facilitated response tendencies during systole have been previously reported specifically for salient stimuli, for example, for fear faces (Ambrosini et al., 2019; Azevedo et al., 2017, 2018; Critchley & Garfinkel, 2015; Garfinkel & Critchley, 2016; Garfinkel et al., 2013, 2014; Park et al., 2013), or stimuli in spatial congruency tasks that trigger automated responses (Larra et al., 2020; von Haugwitz et al., 2024). When stimuli and responses are placed laterally, they create a spatial stimulus-response compatibility which results in speeded responses in case they must be employed at the same location as an appearing stimulus (e.g., left stimulus–left button press; Kornblum et al., 1990; Simon, 1969). We previously found that the cardiac cycle shows behavioral effects for these stimulus-response arrangements increasing response tendencies specifically when stimulus and finger location match (Larra et al., 2020; von Haugwitz et al., 2024). Also in the change detection task we employed here, stimulus and response locations were lateralized and overlapped, which could indicate that the cardiac cycle affects the visuomotor pathway suggested to underlie these spatial congruency effects (Vallesi et al., 2005; Wascher & Wauschkuhn, 1996; Wiegand & Wascher, 2005). Importantly, previous findings reporting increased selection efficiency during systole, that is, less susceptibility to distractors, used central stimuli and response locations where such automated response tendencies are not at play (Pramme et al., 2014, 2016). Thus, the salient orientation change in our task might have been similarly modulated by this pathway. However, as the effect was specific to LOB changes, the behavioral relevance of this mechanism may be constrained predominantly to situations where multiple visual changes and responses compete. A possible source of these effects might be the recently reported modulation of motor processes by the cardiac cycle. Specifically, Paci et al. (2024) found increased intracortical inhibition during systole in the primary motor cortex. Our finding of an enhanced central-posterior lateralization during systole in LOB trials might reflect the allocation of additional premotor resources under higher response selection difficulty in line with increased errors in LOB changes at systolic onsets. By contrast, the attenuated lateralization in systole onsets of LUM trials could point to reduced premotor response encoding during systole when response selection demands are low, in line with the increased missed LUM changes during systole. Given the descriptively earlier onsets of the DEL in LUM systole versus diastole trials however, this could also indicate that in scenarios of low response selection demands the efficiency of response related processes is increased, in line with recent reports on increased motor excitability during systole (Al et al., 2023).

Furthermore, we found an interaction of CPT exposure and cardiac phase on midfrontal theta, and alpha lateralization (LPS). Specifically, in the CPT group, we found that midfrontal theta power was significantly higher during systole compared to diastole following LOB changes (Fig. 7). Moreover, midfrontal theta following LOB changes during diastole was significantly lower in the CPT versus the control group. This decrease in midfrontal theta might be driven by inhibition of regions in the midfrontal cortex following CPT exposure. The CPT elicits sympathetic activation, causing both peripheral release of norepinephrine from sympathetic nerve terminals and epinephrine from the adrenal medulla, increasing central noradrenergic activity which is assumed to inhibit regions in the prefrontal cortex including the anterior cingulate cortex which is suggested to be the generator of midfrontal theta oscillations (Arnsten, 2009, 2015; Asanowicz et al., 2021; Cohen, 2014; Hendriks-Balk et al., 2020; Ishii et al., 1999; LeBlanc et al., 1979; Winer & Carter, 1977). The difference in midfrontal theta between systole and diastole in LOB trials might indicate that the increased errors during systole are driven by an increased response conflict. Kaiser and Schutz-Bosbach (2021) previously showed that midfrontal theta oscillations specifically reflect motor-related adjustments during conflict resolution which could explain why we see an increase in midfrontal theta during systole whereby performance is decreased. For alpha lateralization, we found that the CPT group showed higher negative LPS compared to the control group specifically in systole LUM changes, that is, lower contra- versus ipsilateral alpha power, indicating increased processing of the unilateral luminance change (see Fig. 8; Asanowicz et al., 2021; Bacigalupo & Luck, 2019; Van der Lubbe & Utzerath, 2013). Taken together, this pattern could suggest that reduced midfrontal theta following CPT exposure may result in stronger alpha lateralization as a compensatory mechanism to maintain task-relevant processing during cardiac systole.

Otherwise, the behavioral and electrophysiological effects appeared to be remarkably robust against CPT interventions. The CPT elicits an activation of the medullary pressor response which was confirmed by robust increases in systolic blood pressure in the CPT group, which were absent in the control group. Note, that rather than being induced by the body-warmed water the apparent reduction in heart rate within the control group reflects a simple reset of gradual heart rate increases at the end of each block, that is counteracted by tachycardic effects in the CPT group. Heart rate increases during the CPT are also limited by a second process, that is, baroreflex-mediated deceleration due to peripheral vasoconstriction and consequent increases in blood pressure. Nevertheless, cardiac phase effects were not modulated by exposure to the CPT on the group level. This is in line with previous studies reporting no between-groups modulation of cardiac phase effects after exposure to cardiovascular manipulations (Mussini et al., 2024; von Haugwitz et al., 2024).

On subject level, however, the regression analyses revealed two correlates of cardiac phase effects in LOB errors (Fig. 9). First, we found that HR response to the CPT was negatively associated with the cardiac phase effect. Typically, CPT exposure increases blood pressure, primarily driven by α-adrenergically mediated peripheral vasoconstriction (Frank & Raja, 1994; Lovallo, 1975) and to a lesser extent HR via β-adrenergic stimulation (Houben et al., 1982; Victor et al., 1987). With prolonged CPT exposure, increased baroafferent signaling activates the baroreflex leading to a vagally mediated decrease in HR (Cui et al., 2002; Mancia & Grassi, 1995; Stauss, 2002). Thus, a stronger decrease in HR during the CPT could point to an increased sensitivity to baroafferent signals possibly extending to the cortical level. At the level of the brainstem, this phenomenon is known as baroreflex sensitivity (BRS). While BRS is influenced by a wide range of factors, for example mechanical transduction (carotid/aortic compliance) as well as central neural processing (Cui et al., 2002; Mancia & Grassi, 1995; Stauss, 2002), the observed correlation with heart rate responses suggests that cardiovascular response patterns under load might help explain interindividual variance in susceptibility to cardiac cycle effects. However, to specifically test whether BRS influences cardiac-phase effects, future studies will need to directly derive BRS from beat-to-beat SBP measurements.

Second, we found that HRV moderated the effect of SBP on the phase effect in LOB errors. Specifically, higher HRV was associated with a negative relationship between SBP and the behavioral phase difference, indicating that individuals with lower baroreceptor load during systole (i.e., low SBP) exhibited increased systole versus diastole errors in the LOB condition, particularly under stronger parasympathetic regulation (i.e., high HRV). Descriptively, this effect reversed at low HRV, with SBP positively associated with systole errors. These findings suggest that the observed cardiac phase effect depends on the interplay between baroreceptor load and parasympathetic regulation: behavioral differences between systole and diastole are increased either when parasympathetic regulation is high (i.e., high HRV) and baroreceptor signals are low (i.e., low SBP), or when parasympathetic regulation is low and baroreceptor load is high. Potentially, this effect may also be related to the predictability of interoceptive signals: Al et al. (2020) reported inhibited detection of near‐threshold somatosensory stimuli during systole potentially by the suppression of heartbeat-related firing pattern of afferent neurons in the finger. This effect was more pronounced in individuals with low HRV, suggesting that lower variance in interoceptive signals enhances the precision of such sensory attenuation, or that this effect generally varied with vagal tone.

This study has potential limitations. First, we want to note that we employed regression-based CFA correction (Arnau et al., 2023) and focused primarily on lateralized potentials computed via the double-subtraction approach which effectively removes any CFA. However, we cannot fully exclude that residual CFA generated by the subsequent heartbeat contributed to our CPT theta finding. However, we think the influence of such residual CFA on the effect is rather limited as the observed theta effect is fronto-central, thus topographically distinct from a classic posterior CFA. Furthermore, besides reliably increasing blood pressure, the CPT also elicits pain and emotional responses that may influence perceptual or attentional processing. These additional physiological and psychological effects should be considered when interpreting our findings and while all cardiovascular maneuvers may have their specific side effects, comparing their impact across studies can help to isolate potential cardiovascular drivers.

In summary, cardiac phase systematically modulated change detection: systolic onsets increased errors in spatial-conflict (LOB) changes, biasing responses toward a salient distractor, and increased misses for isolated luminance (LUM) changes. EEG lateralization in a central–posterior premotor cluster (DEL, ~390–500 ms, peak CP3) implicates altered premotor response encoding rather than early sensory gating as the locus of these effects. Although exposure to the CPT elevated blood pressure and heart rate, we found no robust group-level modulation of the cardiac-phase effect; instead, individual differences in HR responses to the CPT as well as an interaction of systolic blood pressure and heart rate variability predicted the magnitude of LOB-phase effects in the CPT group. Together, these results demonstrate that phasic bodily signals can bias visuomotor selection during perceptual conflicts and change detection.

## Supplementary Material

Supplementary Material

## Data Availability

Data and code will be made available upon request after description of research purposes.

## Footnotes

1 We also ran the theta analysis using an alternative baseline window (−400 to −200 ms relative to change onset) to minimize effects of time-frequency smearing. The results remained unchanged, indicating that they are not driven by time-frequency smearing of post-change processes into the pre-stimulus baseline.
