# Supplementary material for: Cardiac phase modulates behavior and response related lateralization in visual spatial conflicts during change detection
Source: Imaging Neurosci (Camb). 2026 Feb 27;4:IMAG.a.1150. doi: 10.1162/IMAG.a.1150 (PMC12951642; doi:10.1162/IMAG.a.1150)
Supplement: Supplementary Material [file IMAG.a.1150_supp.pdf]

Supplemental Material

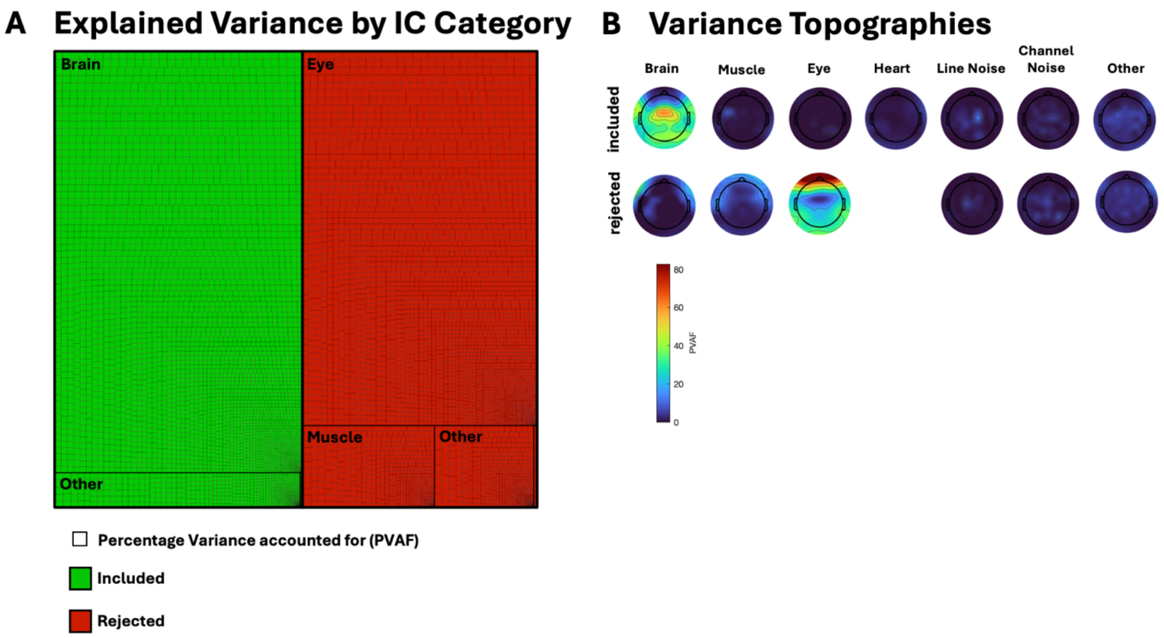

Figure 1. Percentage variance explained (PVAF) by IC category (Panel A) and corresponding topographies (Panel B). Rejected and retained portions of variance are depicted in red and green, respectively. Note, that to calculate PVAF the numerically stable implementation from the Python MNE toolbox (Gramfort et al., 2013) was used, which computes the metric from the ratio of summed squared residuals rather than sample variances; this avoids small rounding errors and spurious negative values while retaining the standard interpretation of explained variance.

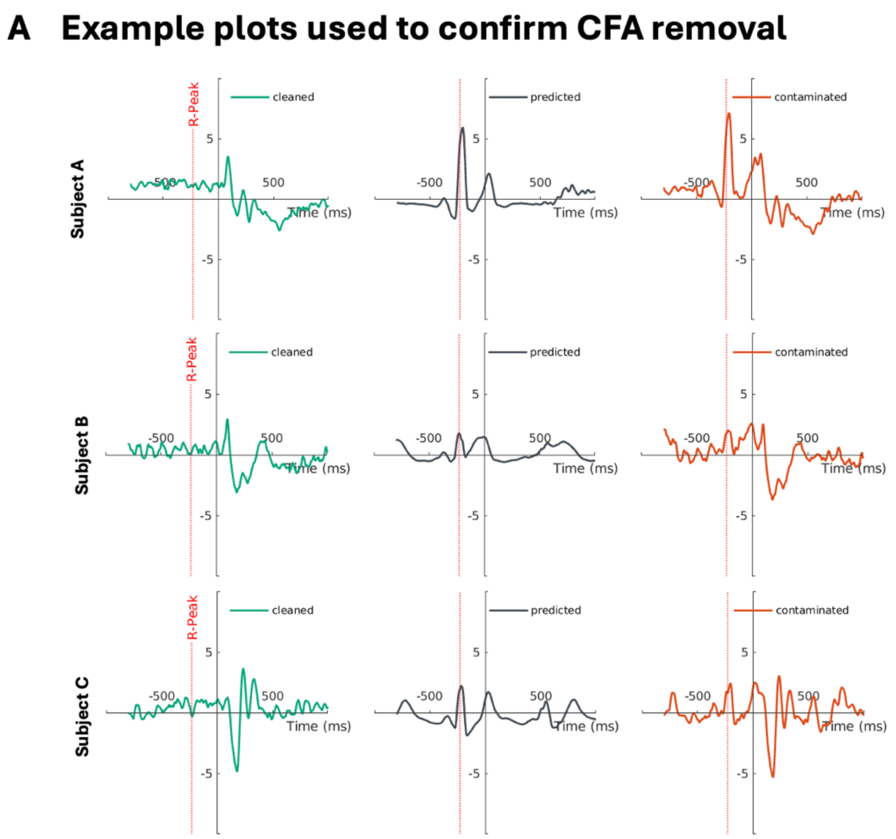

Figure 2. Three examples of single subject plots used for visual confirmation of CFA removal at channel PO7.

Table S1.

Number of trials.

Analysis	*M* (*SD*)
EEG (Accurate)	832.59 (76.81)
Reaction Times; Accuracy	910.04 (63.32)
Errors	45.28 (35.86)
Misses	40.97 (40.78)

Table S2.

Pairwise comparisons of heart rate between CPT and control group across all measurement points.

Time	*t*	*p*	*d*	BF_{10}
Resting Phase	−0.32	.750	1.04	0.26
CPT 1	1.47	.145	−4.79	0.63
Block 1	−0.48	.633	1.56	0.28
**CPT 2**	**2.06**	**.043**	**−6.69**	**1.50**
Block 2	−0.74	.460	2.42	0.32
CPT 3	1.64	.105	−5.33	0.78
Block 3	−0.49	.624	1.60	0.28
CPT 4	1.65	.103	−5.36	0.79
Block 4	−0.38	.704	1.24	0.27

Note. degrees of freedom = 73.

Table S3.

Pairwise comparisons of heart rate between CPT and control group across all measurement points.

Time Point	*t*	*p*	*d*	BF_{10}
Resting Phase	1.14	.260	0.28	0.45
**CPT 1**	**−2.57**	**.012**	**−0.63**	**4.00**
Block 1	1.39	.169	0.34	0.59
**CPT 2**	**−3.92**	**<.001**	**−0.96**	**110**
Block 2	0.96	.342	0.23	0.39
**CPT 3**	**−3.39**	**.001**	**−0.83**	**270**
Block 3	−0.57	.574	−0.14	0.30
**CPT 4**	**−3.68**	**<.001**	**−0.90**	**580**
Block 4	0.71	.480	0.17	0.32

Note. degrees of freedom = 73.

Table S4.

Main Effect of TIME on RMSSD.

Contrast	Estimate	SE	df	*t*	*p*
**RESTING- CPT1**	**-4.15**	**1.35**	**576**	**-3.07**	**.005**
RESTING- BLOCK1	2.06	1.35	576	1.52	.201
**RESTING- CPT2**	**-4.88**	**1.35**	**576**	**-3.61**	**.001**
RESTING- BLOCK2	0.17	1.35	576	0.13	.898
**RESTING- CPT3**	**-4.67**	**1.35**	**576**	**-3.46**	**.002**
RESTING- BLOCK3	-1.98	1.35	576	-1.47	.214
**RESTING- CPT4**	**-5.75**	**1.35**	**576**	**-4.26**	**< .001**
**RESTING- BLOCK4**	**-4.44**	**1.35**	**576**	**-3.29**	**.003**
**CPT1 - BLOCK1**	**6.21**	**1.35**	**576**	**4.60**	**< .001**
CPT1 - CPT2	-0.73	1.35	576	-0.54	.709
**CPT1 - BLOCK2**	**4.32**	**1.35**	**576**	**3.20**	**.004**
CPT1 - CPT3	-0.52	1.35	576	-0.39	.811
CPT1 - BLOCK3	2.17	1.35	576	1.61	.182
CPT1 - CPT4	-1.60	1.35	576	-1.19	.326
CPT1 - BLOCK4	-0.29	1.35	576	-0.22	.898
**BLOCK1 - CPT2**	**-6.93**	**1.35**	**576**	**-5.13**	**< .001**
BLOCK1 - BLOCK2	-1.88	1.35	576	-1.39	.236
**BLOCK1 - CPT3**	**-6.73**	**1.35**	**576**	**-4.98**	**< .001**
**BLOCK1 - BLOCK3**	**-4.04**	**1.35**	**576**	**-2.99**	**.007**
**BLOCK1 - CPT4**	**-7.81**	**1.35**	**576**	**-5.78**	**< .001**
**BLOCK1 - BLOCK4**	**-6.50**	**1.35**	**576**	**-4.81**	**< .001**
**CPT2 - BLOCK2**	**5.05**	**1.35**	**576**	**3.74**	**< .001**
CPT2 - CPT3	0.20	1.35	576	0.15	.898
CPT2 - BLOCK3	2.89	1.35	576	2.14	.065
CPT2 - CPT4	-0.88	1.35	576	-0.65	.641
CPT2 - BLOCK4	0.43	1.35	576	0.32	.843
**BLOCK2 - CPT3**	**-4.85**	**1.35**	**576**	**-3.59**	**.001**
BLOCK2 - BLOCK3	-2.15	1.35	576	-1.60	.182
**BLOCK2 - CPT4**	**-5.93**	**1.35**	**576**	**-4.39**	**< .001**
**BLOCK2 - BLOCK4**	**-4.62**	**1.35**	**576**	**-3.42**	**.002**
CPT3 - BLOCK3	2.69	1.35	576	1.99	.088
CPT3 - CPT4	-1.08	1.35	576	-0.80	.545
CPT3 - BLOCK4	0.23	1.35	576	0.17	.898
**BLOCK3 - CPT4**	**-3.77**	**1.35**	**576**	**-2.79**	**.011**
BLOCK3 - BLOCK4	-2.46	1.35	576	-1.82	.123
CPT4 - BLOCK4	1.31	1.35	576	0.97	.444

Supplementary Results – Change Effects

As displayed in Figure 3 A, the change detection task produced the expected behavioral effects as indicated by significant main effects of CHANGE on RTs (WTS_2 = 114.367, p < .001), accuracy (WTS_3 = 166.451, p < .001), errors (WTS_3 = 126.952, p < .001), as well as misses (WTS_2 = 36.308, p < .001) indicating decreased performance following perceptual conflicts in LOB trials, as well as facilitated performance depending on target salience.

Specifically, reaction times were slowest for LOB changes, compared to both LOU (t(370) = 14.27, p < .001, d_z = 0.74, BF_{10} = 3.5e+24) and LUM changes (t(370) = 11.63, p < .001, d_z = 0.60, BF_{10} = 2.1e+17) while responses to LOU trials were faster than to LUM trials (t(370) = -2.64, p = .009, d_z = -0.14, BF_{10} = 3.5e+02).

Similarly, accuracy was lowest for LOB changes compared to LOU (t(518) = -18.63, p < .001, d_z = -0.82, BF_{10} = 3.7e+33), LUM (t(518) = -18.04, p < .001, d_z = -0.79, BF_{10} = 9.1e+33), and ORI changes (t(518) = -24.55, p < .001, d_z = -1.08, BF_{10} = 1.5e+37). Accuracy in LOU trials did not significantly differ from LUM trials (t(518) = 0.60, p = .551, d_z = 0.03, BF_{10} = 1.6e-01) while it was lower than in ORI trials (t(518) = -5.91, p < .001, d_z = -0.26, BF_{10} = 8e+05). Also, LUM trials showed lower accuracy compared to ORI trials (t(518) = -6.51, p < .001, d_z = -0.29, BF_{10} = 4.7e+8).

Errors were significantly increased for LOB changes compared to LOU (t(518) = 18.97, p < .001, d_z = 0.83, BF_{10} = 1.7e+28), LUM (t(518) = 17.58, p < .001, d_z = 0.77, BF_{10} = 1.6e+29), and ORI changes (t(518) = 15.57, p < .001, d_z = 0.68, BF_{10} = 1.1e+24). LOU changes produced less errors compared to ORI changes (t(518) = -3.39, p = .001, d_z = -0.15, BF_{10} = 2.4e+03). Errors between LOU and LUM trials (t(518) = -1.39, p = .166, d_z = -0.06, BF_{10} = 4.9) as well as LUM and ORI trials did not significantly differ (t(518) = -2.00, p = .055, d_z = -0.09, BF_{10} = 12).

Misses were significantly increased for LOB compared to LOU (t(370) = 8.21, p < .001, d_z = 0.43, BF_{10} = 3e+08) and LUM changes (t(370) = 8.84, p < .001, d_z = 0.46, BF_{10} = 2.3e+08). Misses did not significantly differ between LOU and LUM changes (t(370) = 0.62, p = 0.533, d_z = 0.03, BF_{10} = 1.3e-01).

Table S5.

Cluster based permutation results on group differences in posterior event related lateralization.

	Positive Cluster			Negative Cluster		
CPT-Control	*N*	*N* sig.	*p*	*N*	*N* sig.	*p*
LOB	1	0	0.7938–0.7938	1	0	1.2762–1.2762
LOU	3	0	1.259–1.4122	0	0	
LUM	2	0	0.1022–1.534	1	0	0.8094–0.8094
ORI	4	0	0.781–0.969	0	0	
LOB–SYS	1	0	0.5458–0.5458	0	0	
LOB–DIA	2	0	1.191–1.3726	0	0	
LOU–SYS	0	0		2	0	
LOU–DIA	7	0	0.6142–1.5284	0	0	
LUM–SYS	3	0	0.2556–1.3252	0	0	
LUM–DIA	3	0	0.1286–1.3842	0	0	
ORI–SYS	3	0	0.2154–1.1208	0	0	
ORI–DIA	2	0	0.5594–0.8964	1	0	1.1036–1.1036

Note. All tests used an estimated family-wise $\alpha = .05$; no clusters (positive or negative) reached significance at this level.

Table S6.

CPT-Regression results on cardiovascular drivers of Phase Effect in LOB Errors.

Predictor	b*	SE	*p*	Mdn	90% CI	pd	ROPE
(Intercept)	0.06	0.24	.790	0.09	[-0.27, 0.46]	66.49	32.52
RMSSD [a]	0.40	0.40	.328	0.41	[-0.21, 1.02]	86.80	11.27
SBP [b]	-0.39	0.26	.147	-0.39	[-0.78, 0.01]	94.54	9.02
Δ SBP [c]	0.59	0.34	.097	0.55	[0.03, 1.07]	95.97	5.01
**Δ HR [d]**	**-0.98**	**0.32**	**.006**	**-0.82**	**[-1.31, -0.31]**	**99.48**	**0.00**
HR [e]	-0.22	0.30	.467	-0.23	[-0.68, 0.23]	79.59	19.95
**RMSSD [a] × SBP [b]**	**-0.87**	**0.35**	**.020**	**-0.82**	**[-1.35, -0.28]**	**99.22**	**0.00**
RMSSD [a] × Δ SBP [c]	-0.11	0.57	.851	-0.22	[-1.08, 0.65]	66.42	13.87
SBP [b] × Δ SBP [c]	0.32	0.43	.454	0.42	[-0.23, 1.07]	85.66	11.35
RMSSD [a] × Δ HR [d]	0.38	0.66	.573	0.24	[-0.74, 1.22]	65.63	12.60
SBP [b] × Δ HR [d]	0.25	0.41	.543	0.15	[-0.48, 0.78]	65.82	19.38
Δ SBP [c] × Δ HR [d]	-0.25	0.39	.535	-0.29	[-0.91, 0.32]	78.77	16.04
Δ HR [d] × HR [e]	0.42	0.47	.379	0.27	[-0.45, 0.97]	73.51	15.17
RMSSD [a] × SBP [b] × Δ SBP [c]	0.85	0.56	.144	0.82	[-0.04, 1.68]	94.16	4.22
RMSSD [a] × SBP [b] × Δ HR	1.02	0.71	.167	0.96	[-0.11, 2.02]	93.14	3.73
RMSSD [a] × Δ SBP [c] × Δ HR	-0.79	0.77	.318	-0.52	[-1.64, 0.62]	77.94	8.48
SBP [b] × Δ SBP [c] × Δ HR [d]	0.63	0.50	.218	0.45	[-0.32, 1.2]	83.99	10.35
RMSSD [a] × SBP [b] × Δ SBP [c] × Δ HR [d]	0.35	1.33	.795	0.04	[-1.79, 1.89]	51.35	7.15

Note. Robust model = MM-estimator via lmrob; Bayesian model = brms with student-t priors. (R^2 = .582, RSE = .646; Bayesian R^2 = .528, 95% CI [.381, .628]). Bayesian results show posterior median and 90% credible intervals. [a] Mean RMSSD (Root Mean Square of Successive Differences) during experimental blocks. [b] Systolic Blood Pressure (SBP) measures at the end of experimental blocks. [c] Mean SBP responses to CPT [CPT-Resting]. [d] Mean heart rate (HR) responses to the CPT [CPT-Resting]. [e] Mean HR during Blocks.

Table S7.
Regression results on cardiovascular drivers of Phase Effect in LOB RIDE response cluster.

Predictor	*b**	SE	*p*	Mdn	90% CI	pd	ROPE
(Intercept)	-0.31	0.34	0.372	-0.27	[-0.81, 0.28]	79.61	18.76
RMSSD [a]	-0.24	0.58	0.688	-0.17	[-1.05, 0.74]	61.98	15.32
SBP [b]	0.20	0.37	0.604	0.10	[-0.5, 0.69]	61.49	23.37
Δ SBP [c]	-0.10	0.48	0.841	-0.09	[-0.83, 0.66]	58.17	19.49
Δ HR [d]	0.08	0.46	0.856	0.16	[-0.56, 0.88]	64.74	19.00
HR [e]	-0.28	0.42	0.509	-0.23	[-0.88, 0.43]	72.32	18.30
RMSSD [a] × SBP [b]	-0.23	0.51	0.651	-0.14	[-0.91, 0.63]	62.06	17.86
RMSSD [a] × Δ SBP [c]	-0.34	0.82	0.678	-0.39	[-1.65, 0.84]	69.84	10.27
SBP [b] × Δ SBP [c]	-0.59	0.61	0.341	-0.50	[-1.42, 0.45]	81.06	10.56
RMSSD [a] × Δ HR [d]	0.71	0.94	0.459	0.60	[-0.77, 2.02]	76.70	8.04
SBP [b] × Δ HR [d]	0.98	0.60	0.114	0.79	[-0.13, 1.69]	92.26	5.61
Δ SBP [c] × Δ HR [d]	-0.03	0.57	0.964	-0.06	[-0.95, 0.82]	54.31	16.88
Δ HR [d] × HR [e]	0.06	0.67	0.929	-0.06	[-1.09, 0.97]	53.99	14.70
RMSSD [a] × SBP [b] × Δ SBP [c]	0.72	0.81	0.382	0.52	[-0.74, 1.76]	75.96	9.03
RMSSD [a] × SBP [b] × Δ HR	-1.28	1.03	0.226	-0.96	[-2.47, 0.58]	84.82	5.39
RMSSD [a] × Δ SBP [c] × Δ HR	-0.61	1.10	0.584	-0.35	[-1.92, 1.21]	64.28	8.55
SBP [b] × Δ SBP [c] × Δ HR [d]	0.38	0.72	0.605	0.25	[-0.87, 1.35]	64.68	12.85
RMSSD [a] × SBP [b] × Δ SBP [c] × Δ HR [d]	0.97	1.91	0.614	0.25	[-2.21, 2.71]	57.27	6.23

Note. Robust model = MM-estimator via lmrob; Bayesian model = brms with student-t priors. (R^2 = .232, RSE = 1.15; Bayesian R^2 = .341, 95% CI [.215, .448]). Bayesian results show posterior median and 90% credible intervals. [a] Mean RMSSD (Root Mean Square of Successive Differences) during experimental blocks. [b] Systolic Blood Pressure (SBP) measures at the end of experimental blocks. [c] Mean SBP responses to CPT [CPT-Resting] [d] Mean heart rate (HR) responses to the CPT [CPT-Resting]. [e] Mean HR during Blocks.

Table S8.
Regression results on cardiovascular drivers of Phase Effect in LOB Theta cluster.

Predictor	b*	SE	*p*	Mdn	90% CI	pd	ROPE
(Intercept)	0.11	0.14	0.427	0.12	[-0.1, 0.33]	81.84	17.81
RMSSD [a]	-0.01	0.24	0.974	-0.02	[-0.4, 0.36]	53.56	15.70
SBP [b]	-0.05	0.15	0.732	-0.05	[-0.28, 0.19]	63.07	23.86
Δ SBP [c]	-0.11	0.20	0.604	-0.13	[-0.44, 0.18]	75.49	15.13
Δ HR [d]	0.16	0.19	0.403	0.17	[-0.13, 0.47]	83.49	12.38
HR [e]	0.20	0.18	0.280	0.19	[-0.08, 0.47]	87.60	10.63
RMSSD [a] × SBP [b]	0.16	0.21	0.445	0.17	[-0.16, 0.49]	80.27	12.61
RMSSD [a] × Δ SBP [c]	-0.09	0.34	0.804	-0.09	[-0.63, 0.43]	61.66	11.05
SBP [b] × Δ SBP [c]	-0.09	0.25	0.713	-0.10	[-0.5, 0.3]	65.86	13.83
RMSSD [a] × Δ HR [d]	-0.13	0.40	0.741	-0.09	[-0.7, 0.51]	59.38	9.78
SBP [b] × Δ HR [d]	-0.03	0.25	0.915	-0.01	[-0.4, 0.37]	52.28	15.68
Δ SBP [c] × Δ HR [d]	0.07	0.24	0.787	0.08	[-0.29, 0.45]	64.55	14.96
Δ HR [d] × HR [e]	-0.05	0.28	0.857	-0.03	[-0.46, 0.4]	54.83	13.67
RMSSD [a] × SBP [b] × Δ SBP [c]	-0.43	0.33	0.201	-0.43	[-0.95, 0.08]	92.03	4.16
RMSSD [a] × SBP [b] × Δ HR	0.46	0.43	0.288	0.46	[-0.18, 1.12]	88.49	4.30
RMSSD [a] × Δ SBP [c] × Δ HR	0.30	0.46	0.525	0.29	[-0.41, 0.98]	75.66	6.54
SBP [b] × Δ SBP [c] × Δ HR [d]	0.14	0.30	0.650	0.12	[-0.34, 0.59]	67.77	11.66
RMSSD [a] × SBP [b] × Δ SBP [c] × Δ HR [d]	-0.20	0.78	0.800	-0.18	[-1.34, 0.97]	60.92	5.09

Note. Robust model = MM-estimator via lmrob; Bayesian model = brms with student-t priors. (R^2 = .29, RSE = 0.372; Bayesian R^2 = .397, 95% CI [.263, .502]). Bayesian results show posterior median and 90% credible intervals. [a] Mean RMSSD (Root Mean Square of Successive Differences) during experimental blocks. [b] Systolic Blood Pressure (SBP) measures at the end of experimental blocks. [c] Mean SBP responses to CPT [CPT-Resting] [d] Mean heart rate (HR) responses to the CPT [CPT-Resting]. [e] Mean HR during Blocks.

Table S9.

Regression results on cardiovascular drivers of Phase Effect in LUM Misses.

Predictor	b*	SE	*p*	Mdn	90% CI	pd	ROPE
(Intercept)	0.25	0.26	0.350	0.20	[-0.24, 0.64]	77.84	20.28
RMSSD [a]	0.00	0.44	0.998	-0.07	[-0.8, 0.67]	56.19	16.56
SBP [b]	0.08	0.28	0.782	0.09	[-0.39, 0.56]	62.00	24.21
Δ SBP [c]	-0.03	0.40	0.946	-0.06	[-0.7, 0.58]	55.88	18.82
Δ HR [d]	0.32	0.42	0.446	0.43	[-0.23, 1.11]	86.32	10.01
HR [e]	0.25	0.33	0.463	0.22	[-0.34, 0.77]	74.51	17.55
RMSSD [a] × SBP [b]	-0.04	0.43	0.922	-0.01	[-0.73, 0.69]	51.26	17.20
RMSSD [a] × Δ SBP [c]	0.15	0.71	0.839	0.08	[-1.04, 1.2]	55.19	11.03
SBP [b] × Δ SBP [c]	-0.35	0.49	0.485	-0.24	[-1.04, 0.57]	69.47	13.58
RMSSD [a] × Δ HR [d]	0.15	0.89	0.865	0.32	[-1.06, 1.7]	65.09	7.93
SBP [b] × Δ HR [d]	0.18	0.53	0.732	0.12	[-0.73, 0.97]	59.42	13.96
Δ SBP [c] × Δ HR [d]	-0.56	0.44	0.217	-0.54	[-1.26, 0.19]	89.22	7.61
Δ HR [d] × HR [e]	0.40	0.51	0.435	0.35	[-0.48, 1.18]	76.17	11.13
RMSSD [a] × SBP [b] × Δ SBP [c]	-1.68	0.97	0.097	-1.28	[-2.78, 0.23]	91.82	2.98
RMSSD [a] × SBP [b] × Δ HR	1.65	1.13	0.157	1.23	[-0.49, 2.96]	88.19	3.53
RMSSD [a] × Δ SBP [c] × Δ HR	-0.03	0.84	0.973	-0.04	[-1.34, 1.28]	51.82	9.56
SBP [b] × Δ SBP [c] × Δ HR [d]	-0.22	0.59	0.710	-0.31	[-1.26, 0.63]	70.91	11.14
RMSSD [a] × SBP [b] × Δ SBP [c] × Δ HR [d]	0.69	1.51	0.651	0.38	[-1.75, 2.56]	61.76	5.40

Note. Robust model = MM-estimator via lmrob; Bayesian model = brms with student-t priors. (R^2 = .306, RSE = 0.94; Bayesian R^2 = .376, 95% CI [.245, .484]). Bayesian results show posterior median and 90% credible intervals. [a] Mean RMSSD (Root Mean Square of Successive Differences) during experimental blocks. [b] Systolic Blood Pressure (SBP) measures at the end of experimental blocks. [c] Mean SBP responses to CPT [CPT-Resting] [d] Mean heart rate (HR) responses to the CPT [CPT-Resting]. [e] Mean HR during Blocks.

Table S10.
Regression results on cardiovascular drivers of Phase Effect in LUM RIDE response cluster.

Predictor	b*	SE	*p*	Mdn	90% CI	pd	ROPE
(Intercept)	-0.08	0.24	.731	0.08	[-0.34, 0.51]	63.01	31.64
RMSSD [a]	0.23	0.40	.578	0.36	[-0.34, 1.08]	80.27	14.07
SBP [b]	-0.44	0.26	.097	-0.49	[-0.94, -0.04]	96.40	5.35
Δ SBP [c]	0.46	0.36	.216	0.49	[-0.12, 1.11]	90.90	9.33
Δ HR [d]	0.21	0.37	.574	0.20	[-0.44, 0.83]	69.94	19.66
HR [e]	-0.14	0.31	.667	0.06	[-0.49, 0.62]	57.04	25.38
RMSSD [a] × SBP [b]	-0.16	0.38	.687	-0.08	[-0.74, 0.58]	58.56	21.16
RMSSD [a] × Δ SBP [c]	-1.14	0.63	.081	-1.11	[-2.2, -0.02]	95.27	3.08
SBP [b] × Δ SBP [c]	0.51	0.47	.290	0.16	[-0.62, 0.95]	63.26	17.17
RMSSD [a] × Δ HR [d]	0.50	0.80	.533	0.29	[-1.05, 1.62]	64.72	10.13
SBP [b] × Δ HR [d]	-0.32	0.48	.516	-0.13	[-0.92, 0.7]	59.99	16.88
Δ SBP [c] × Δ HR [d]	-0.77	0.40	.071	-0.94	[-1.65, -0.22]	98.31	0.33
Δ HR [d] × HR [e]	-0.49	0.44	.282	-0.47	[-1.26, 0.32]	83.93	10.87
RMSSD [a] × SBP [b] × Δ SBP [c]	-1.25	0.85	.156	-0.91	[-2.33, 0.56]	85.14	5.65
RMSSD [a] × SBP [b] × Δ HR	1.24	1.00	.228	1.01	[-0.66, 2.65]	84.50	4.90
RMSSD [a] × Δ SBP [c] × Δ HR	0.27	0.74	.716	0.39	[-0.88, 1.69]	69.40	9.87
SBP [b] × Δ SBP [c] × Δ HR [d]	-0.37	0.54	.495	-0.35	[-1.25, 0.58]	73.69	12.75
RMSSD [a] × SBP [b] × Δ SBP [c] × Δ HR [d]	-0.33	1.32	.808	-0.26	[-2.32, 1.79]	58.33	7.02

Note. Robust model = MM-estimator via lmrob; Bayesian model = brms with student-t priors. (R^2 = .585, RSE = 0.633; Bayesian R^2 = .481, 95% CI [.328, .591]). Bayesian results show posterior median and 90% credible intervals. [a] Mean RMSSD (Root Mean Square of Successive Differences) during experimental blocks. [b] Systolic Blood Pressure (SBP) measures at the end of experimental blocks. [c] Mean SBP responses to CPT [CPT-Resting] [d] Mean heart rate (HR) responses to the CPT [CPT-Resting]. [e] Mean HR during Blocks.
